# Adipose Tissue Epigenetic Profile in Obesity-Related Dysglycemia - A Systematic Review

**DOI:** 10.3389/fendo.2021.681649

**Published:** 2021-06-29

**Authors:** Sara Andrade, Tiago Morais, Ionel Sandovici, Alexandre L. Seabra, Miguel Constância, Mariana P. Monteiro

**Affiliations:** ^1^ Endocrine and Metabolic Research, Unit for Multidisciplinary Research in Biomedicine (UMIB), University of Porto, Porto, Portugal; ^2^ Department of Anatomy, Institute of Biomedical Sciences Abel Salazar (ICBAS), University of Porto, Porto, Portugal; ^3^ University of Cambridge Metabolic Research Laboratories and MRC Metabolic Diseases Unit, Institute of Metabolic Science, Addenbrookes Hospital, Cambridge, United Kingdom; ^4^ Department of Obstetrics and Gynaecology and National Institute for Health Research Cambridge Biomedical Research Centre, Cambridge, United Kingdom; ^5^ Centre for Trophoblast Research, Department of Physiology, Development and Neuroscience, University of Cambridge, Cambridge, United Kingdom; ^6^ National Institute of Health Research, Cambridge Biomedical Research Centre, Cambridge, United Kingdom

**Keywords:** adipose tissue, obesity, dysglycemia, type 2 diabetes, insulin resistance, DNA methylation, histone modifications, long non-coding RNAs

## Abstract

**Background:**

Obesity is a major risk factor for dysglycemic disorders, including type 2 diabetes (T2D). However, there is wide phenotypic variation in metabolic profiles. Tissue-specific epigenetic modifications could be partially accountable for the observed phenotypic variability.

**Scope:**

The aim of this systematic review was to summarize the available data on epigenetic signatures in human adipose tissue (AT) that characterize overweight or obesity-related insulin resistance (IR) and dysglycemia states and to identify potential underlying mechanisms through the use of unbiased bioinformatics approaches.

**Methods:**

Original data published in the last decade concerning the comparison of epigenetic marks in human AT of individuals with metabolically unhealthy overweight/obesity (MUHO) versus normal weight individuals or individuals with metabolically healthy overweight/obesity (MHO) was assessed. Furthermore, association of these epigenetic marks with IR/dysglycemic traits, including T2D, was compiled.

**Results:**

We catalogued more than two thousand differentially methylated regions (DMRs; above the cut-off of 5%) in the AT of individuals with MUHO compared to individuals with MHO. These DNA methylation changes were less likely to occur around the promoter regions and were enriched at loci implicated in intracellular signaling (signal transduction mediated by small GTPases, ERK1/2 signaling and intracellular trafficking). We also identified a network of seven transcription factors that may play an important role in targeting DNA methylation changes to specific genes in the AT of subjects with MUHO, contributing to the pathogeny of obesity-related IR/T2D. Furthermore, we found differentially methylated CpG sites at 8 genes that were present in AT and whole blood, suggesting that DMRs in whole blood could be potentially used as accessible biomarkers of MUHO.

**Conclusions:**

The overall evidence linking epigenetic alterations in key tissues such AT to metabolic complications in human obesity is still very limited, highlighting the need for further studies, particularly those focusing on epigenetic marks other than DNA methylation. Our initial analysis suggests that DNA methylation patterns can potentially discriminate between MUHO from MHO and provide new clues into why some people with obesity are less susceptible to dysglycemia. Identifying AT-specific epigenetic targets could also lead to novel approaches to modify the progression of individuals with obesity towards metabolic disease.

**Systematic Review Registration:**

PROSPERO, identifier CRD42021227237.

## Introduction

Obesity is a complex multifactorial disease that results from the interplay between environmental and genetic factors (1). Overweight and obesity are characterized by adipose tissue (AT) expansion, attributed either to hyperplasia and/or hypertrophy, which eventually becomes dysfunctional (2). AT expansion is often associated with a chronic low-grade inflammatory state that will increase oxidative stress (3) and may have a significant contribution for obesity-related comorbidities development, including insulin resistance (IR) and dysglycemia (4).

Dysglycemia states are defined as conditions of abnormal glucose homeostasis and include impaired fasting glucose, impaired glucose tolerance or both. Fasting plasma glucose above 100 mg/dl and 126 mg/dl (5.6 mmol/L and 7.0 mml/L) or hemoglobin A1c (HbA1c) above 5.7% and 6.5% are used as biochemical thresholds to define the clinical conditions known as pre-diabetes and diabetes, respectively (5). Type 2 diabetes (T2D) is one of the most prevalent comorbidities among individuals with obesity, being responsible for a great number of adverse health outcomes (6). Dysglycemic conditions are characterized by a continuous spectrum of glycemic imbalance (5). From a pathological perspective, these processes are initiated by increased resistance to insulin action in peripheral organs, such as skeletal muscle, liver and AT (7). Consequent to a decreased insulin-mediated glucose uptake, the pancreas is stimulated to increase insulin secretion in an attempt to overcome resistance (7, 8). When the pancreatic capacity to sustain insulin hypersecretion is lost, circulating glucose levels rise and pre-diabetes or overt T2D arise (7, 8).

Individuals with severe obesity can be defined as metabolically healthy obese (MHO), i.e. if no evidence of abnormal metabolic parameters is found in routine clinical and biochemical assessments (9). However, a considerable number of patients with obesity are also affected by obesity-related metabolic disorders, such as pre-diabetes, T2D, hypertension and dyslipidemia, and therefore are clinically classified as having metabolic unhealthy obesity (MUHO) (10). For the purpose of this systematic review, only studies that sought to evaluate the differences between individuals with overweight or obesity in the absence or presence of dysglycemia, were considered and defined as MHO or MUHO, respectively.

The relationship between increasing body mass index (BMI) above 25 kg/m^2^ or 30 kg/m^2^, which is the anthropometric measurement most frequently used to define overweight and obesity, except for Asian ethnic background populations, and T2D is far from being linear. Obesity is a well-recognized risk factor for T2D. The risk of developing T2D increases with increasing BMI when compared to normal weight individuals (10). However, despite approximately 90% of the patients with T2D presenting concomitant overweight or obesity (11), less than one third of the patients with severe obesity harbor T2D as comorbid condition (12). Therefore, the relationship between obesity and T2D is a complex one. Moreover, the risk of cardiovascular disease and cardiovascular disease mortality, which is the number one cause of death among individuals with obesity, increases not only with increasing BMI, but also with the number of metabolic abnormalities, being the highest among patients with T2D (13). Thus, the presence of T2D renders a more advanced disease stage to obesity, forecasting a poorer prognosis and decreased life expectancy (13). Identifying the triggering factors responsible for T2D development in some individuals with obesity is clinically very relevant, because it would allow to implement targeted intervention strategies with the aim of preventing cardiometabolic complications in those patients at higher risk

T2D is a multifactorial and polygenic disease with hundreds of genetic variants identified as risk factors in genome-wide association studies (GWAS) (14). Despite the fact that obesity and T2D can unquestionably be associated as mentioned above, only a few loci were identified as being related with both conditions, such as *FTO*, *MC4R*, *ADAMTS9*, *GRB14*/*COBLL1* and *QPCTL*/*GIPR* (11), suggesting that these genes may have a particularly important role in the pathogeny of both conditions. However, it is important to notice that single nucleotide polymorphisms (SNPs) primarily associated with obesity tend to have a positive correlation between the effect size on BMI and the effect of the same SNP on T2D, yet SNPs primarily associated with T2D have no impact on BMI *per se* (15). Importantly, both GWAS and gene candidate approaches have led to the identification of genes implicated in the pathogenesis of obesity and/or T2D (14, 16), which are involved in critical pathways for glucose regulatory processes, such as insulin signaling (*INSR*, *IRS1*, *IRS2*) (16–20), beta-cell differentiation and insulin secretion (*PDX1*, *HNF4A*, *TCF7L2*, *SLC2A4*, *GLP1R*, *KCNQ1*) (16, 17, 21–24), adipocyte differentiation (*PPARG*, *PPARGC1A*, *LEP*, *ADIPOQ*) (17, 19, 20, 25, 26), mitochondrial biogenesis and function (*PGC1*) (27), lipid and glucose homeostasis (*SREBF1*) (28) and cytokine signaling and inflammation (*ADIPOQ*) (29).

Previous studies also sought to identify genetic determinants that could explain the diversity in metabolic phenotypes among patients with overweight or obesity. Genetic risk scores derived from GWAS (30) resulted in modest improvements in the accuracy to predict T2D compared to traditional risk scores that rely on patient characteristics, namely age, family history of T2D and BMI, such as the FINDRISK Diabetes Score (31). Few studies focused on MHO and MUHO, mainly due to difficulties in patient characterization. It appears that there is no clear association between susceptibility to develop obesity-associated comorbidities and adiposity related genes, with some variants of these genes having potentially cardiometabolic protective effects (*IRS1*, *COBLL1*/*GRB14*, *PLA2G6* and *TOMM40*) (32). The lack of a clear association may be explained by the observation that within the same gene some variants could have opposing effects on these traits (e.g. *COBLL1*/*GRB14)* (32). However, most recently, the first genome-wide cross-phenotype meta-analysis of adiposity–cardiometabolic trait pairs led to the discovery of 62 loci, clustered in three groups, of which the same allele was significantly associated with both higher adiposity and lower cardiometabolic risk (33).

Additional factors known to modulate gene expression, such as epigenetic marks, were hypothesized to play a relevant role in the pathogenesis of obesity-related dysglycemia (34). The term epigenetics refers to reversible alterations in gene activity, which occur without altering the DNA sequence and that are heritable (during cell divisions in somatic cells, but also through the germline and inherited trans-generationally) (35). Epigenetic information is laid upon the genome as epigenetic marks that include DNA methylation, histone modifications (such as methylation, acetylation, etc.), non-coding RNAs and chromatin remodelers, all of which affect the chromatin architecture and provide long-term stability of gene expression patterns (35). Epigenetic states are tissue-specific and are implicated in a range of cellular processes, such as cell differentiation, genomic imprinting and chromosome X-inactivation in females (34). Importantly, epigenetic modifications are able to influence the cellular phenotype and responsiveness to external stimuli (36).

Despite the fact that an association of epigenetics with obesity and dysglycemic disorders has been known since the nineties, the interest in epigenetic alterations associated with obesity and obesity-related dysglycemic disorders has increased exponentially in the last decade (37). Several studies have focused on identifying epigenetic modifications occurring in patients with obesity and dysglycemia states including T2D, when compared to normal weight (NW) and/or individuals with normoglycemia, thus providing considerable advance in our knowledge (38, 39). In fact, numerous DNA differentially methylated regions (DMRs) associated with T2D were identified in targeted or epigenome-wide association studies (EWAS), some of which occurred in genes reported to be of particular interest given the direct involvement in mechanisms known to regulate glucose homeostasis (40). Consistently, increased DNA methylation in *PPARGC1A* was found to down-regulate gene expression in islet cells (41), skeletal muscle (42) and AT of individuals at high risk for T2D (43), along with decreased mitochondrial content (44). Moreover, *PPARGC1A* methylation was shown to be highly responsive to exercise (45) and bariatric surgery (42). Additionally, altered DNA methylation in *ABCG1* and *SREBF1* genes, involved in lipid homeostasis, were demonstrated to be associated with down-regulation of mRNA levels in the liver and skeletal muscle from individuals with T2D (46). These are only a few among the large number of genes in which epigenetic changes were reported to be correlated with glucose and insulin concentrations, BMI and Homeostatic Model Assessment of Insulin Resistance (HOMA-IR) and were identified as being associated with the risk of future T2D (40, 47).

Nevertheless, the role of epigenetic modifications in modulating the risk for obesity-related dysglycemic disorders is far from being fully understood. The type, strength, and rate of epigenetic changes and how cell-specific these are, or which individuals are more predisposed to develop it, remains to be elucidated. This is partially because human data so far available, with very few exceptions, is mostly derived from small patient cohorts, or limited to whole blood (WB) analysis, although many epigenetic modifications are known to be tissue-specific (48). Therefore, since AT expansion and dysfunction are known to play a crucial role in the development of several obesity-associated metabolic comorbidities, gaining knowledge on the AT-specific epigenetic modifications is paramount for understanding the mechanisms of disease, which could lead to the identification of potential targets for treatment intervention. Importantly, the visceral adipose tissue (VAT) is recognized for having a higher metabolic activity compared to the subcutaneous adipose tissue (SAT), as well as by having a greater impact on systemic metabolism *via* rapid release of free fatty acids (49, 50). Additionally, the interplay of altered metabolic and physiological states within distinct AT territories, such as VAT and SAT, were demonstrated to be detrimental for whole-body glucose homeostasis (49). This evidence reinforces the pressing need to evaluate and assess the relative contributions of distinct epigenetic modifications observed in each AT depot in the mechanisms associated with IR and dysglycemic states. Therefore, the aim of this systematic review was to summarize the available data on ‘epigenetic fingerprints’ in human AT that allow differentiation between MHO and MUHO associated with dysglycemia states, and to uncover the potential contribution of epigenetic-regulated cellular pathways. In addition, we discuss the major drawbacks of the current human models for the study of epigenetics in AT and the limitations of epigenetic analyses in this tissue and point out to future directions of research.

## Materials and Methods

### Literature Search

Publications reporting original data focused on associations between epigenetic marks, namely DNA methylation, histone modifications and long non-coding RNAs (lncRNA) in AT of subjects with MUHO were searched on three different databases – PubMed, Scopus and Web of Science – in December 2020. The search was limited to human studies, written in English and published over the last 10 years. The search strategies for the different databases are reported in the **Supplementary Table 1**.

**Figure 1:**
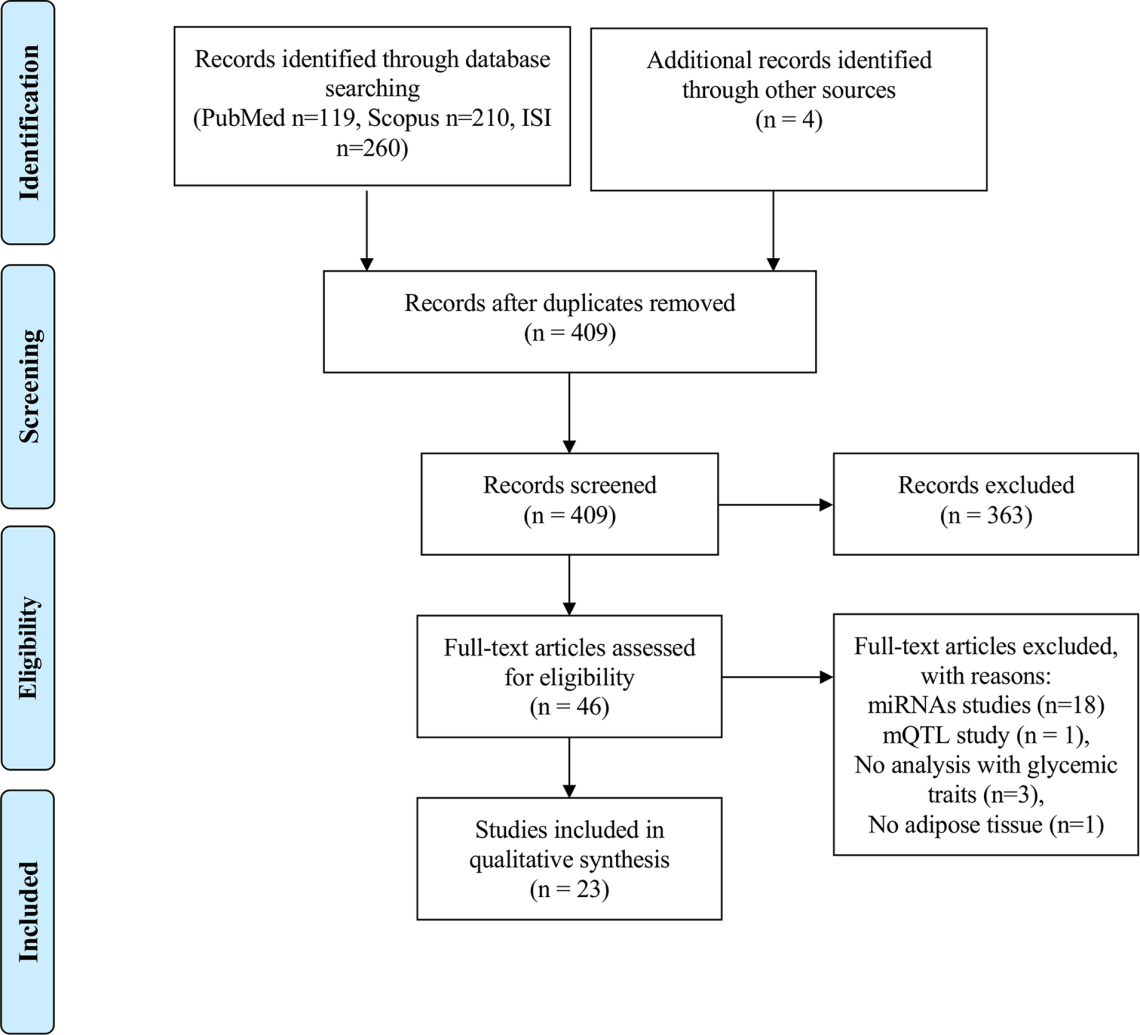
PRISMA flowchart on the literature selection process.

Our search strategy identified 119 papers in PubMed, 210 in Scopus and 260 in Web of Science, resulting in a total of 589 papers for an initial screen. Furthermore, 4 papers were identified from reading the reference list of papers on the topic, which were not retrieved from any of the above-mentioned database searches. After eliminating duplicates, a total number of 409 papers were independently screened by three researchers (ALS, TM and SA) by reading the titles in the first instance. Discordances were resolved with the aid of a fourth researcher (MPM). Papers reporting on epigenetic studies in human AT in individuals with MUHO, or individuals with obesity associated with glycemic traits were considered relevant for the scope of this review and further selected for full-text evaluation (n=46). After reading the full texts, an additional number of 23 papers were eliminated due to the following reasons: miRNAs studies (n=18), DNA methylation quantitative trait locus (mQTL) study (n=1), no data on subjects with altered glycemic traits (n=3), no AT (n=1) (**Figure 1**). Risk of Bias was evaluated for every study by using a modified Newcastle-Ottawa Scale for cross sectional studies (51) (**Supplementary Table 2**). The protocol for this systematic review was registered in PROSPERO (CRD42021227237) and the PRISMA Statement guidelines were used for the reporting of the findings.

Amongst the 23 papers included in this systematic review, 19 papers reported studies on DNA methylation, 2 papers presented data on histone modifications and 2 papers conducted studies on lncRNAs. Of the 19 papers that reported data on DNA methylation in AT from individuals with MUHO, 8 papers performed targeted epigenome studies (52–59) and 11 papers reported genome-wide studies (60–70). The main findings on the targeted DNA methylation studies are summarized in **Table 1**.

**Table 1:** Data obtained from targeted studies assessing DNA methylation in dysglycemic states or correlated with glycemic parameters.

Reference	Studied groups for DNA methylation analysis	Sex distribution of respective groups	Ages of respective groups (years)	Experimental design (studied glycemic parameters)	Tissue	Genes	Main Findings (NW/MHO *vs* MUHO)
							*ADIPOQ*
							Subjects with MUHO had higher promoter gene methylation frequency when compared with subjects with MHO
				Cross-sectional			There is a positive association between methylation and fasting glucose
Zhang J. et al. (59)	Uygur subjects	MHO=44 F/4 M	MHO=45.94 ± 10.01	Case-control (MHO *vs* MUHO; fasting glucose)	VAT (Total)	*TNFA*, *ADIPOQ, MCP1*	*TNFA*
	MHO (n=48),	MUHO=10 F/16 M	MUHO=55.25 ± 12.48				Subjects with MUHO had lower promoter gene methylation frequency when compared with subjects with MHO
	MUHO (n= 26)						There is a negative association between methylation and fasting glucose
							*MCP1*
							No differences between groups were found in promoter methylation frequency
Houde, A. A. et al. (53)	MHO (n=73)	40 F/33 M	34.7 ± 7.1	Cross-sectional (fasting glucose)	VAT (Total) SAT (Total)	*LEP*, *ADIPOQ*	*LEP* and *ADIPOQ* No association between CpGs methylation near the proximal promoter of *LEP* or *ADIPOQ* gene locus and fasting glucose levels in subjects with MHO
Main A. M. et al. (55)	Danish subjects from the EUGENE2 Consortium MHO (n=87), MUHO (n=50)	MHO=61 F/26 M MUHO=25 F/25 M	F=53.9 ± 10.7 M=53.8 ± 12.0	Cross-sectional Case-control (MHO *vs* MUHO)	SAT (Total)	*HIF3A*	*HIF3A* There were no significant differences in intergenic DNA methylation levels between subjects with MUHO and subjects with MHO

Willmer T. et al. (57)	Female South African subjects	54 F	22-36	Cross-sectional Case-control	SAT (Total)	*GR*, *FKBP5*	*GR* No correlations between DNA methylation and glycemic traits
	(n=54)			(glucose, insulin, insulin sensitivity and HOMA-IR)			
	NW, MHO, MUHO (BMI 21.7 – 41.6)						*FKBP5* Positive correlations were found for fasting insulin/HOMA-IR and DNA methylation In gluteal SAT, gene expression (qRT-PCR) is negatively correlated with methylation levels, fasting insulin and HOMA-IR and it is positively correlated with insulin sensitivity
							Global methylation in VAT is associated with obesity but not with T2D; is positively correlated with HOMA-IR and negatively correlated with QUICKI
							*DNMT3a*
							Expression is increased in subjects with MUHO and is also positively correlated with global DNA methylation
Małodobra-Mazur M. et al. (56)	NW (n=26)	NW=9 F/17 M	NW=47 ± 15	Cross-sectional Case-control (MHO *vs* MUHO) (HOMA-IR, QUICKI)	VAT (Total)	Global Methylation, *INSR*, *PIK3R1*, *SLC2A4*	*INSR* and *SCL2A4*
	MUHO (n=9)	MUHO=6 F/3 M	MUHO=52 ± 10				Promoter methylation is increased in subjects with MUHO
							qRT-PCR analysis showed a decrease in mRNA expression in subjects with MUHO
							Promoter methylation is positively correlated with HOMA-IR and negatively correlated with QUICKI For *SCL2A4*, promoter methylation is also negatively correlated with *SCL2A4* gene expression
							*PIK3R1*
							qRT-PCR analysis showed a decrease in mRNA expression
You D. et al. (58)	(A)BMI and T2D discordant MZ twin pairs from Scandinavian twin registries MHO (n=14) MUHO (n=14) (B)MHO (n=28) MUHO (n= 28)	(A)MHO=5 F/9 M MUHO=5 F/9 M (B)MHO=13 F/15 M MUHO=13 F/15 M	(A)MHO=67.6 ± 7.7 MUHO=67.6 ± 7.7 (B)MHO=74.3 ± 4.3 MUHO =74.5 ± 4.2				
				Cross-sectional Case-control (MHO *vs* MUHO)	SAT (Total)	*FGF21*	*FGF21* Hypermethylation of four CpG sites (differences of 3.5%, 3.0%, 3.7% and 2.3% annotated t*o FGF*21 in subjects with MUHO compared with controls)
							A significant negative correlation was found between DNA methylation in 1 CpG and mRNA expression of *FGF21*
Castellano-Castillo D. et al. (52)	MHO/MUHO (n=60)	47 F/23 M	(41.53 ± 9.78) -	Cross-sectional Case-control (glucose, insulin and HOMA-IR)	VAT (Total)	*C3*	*C3*
			(56.70 ± 15.24)				No correlations between DNA methylation and HOMA-IR or glucose were found
Krause C. et al. (54)	German cohort			Cross-sectional Case-control (MHO *vs* MUHO) (blood glucose, HbA1c, HOMA-IR)			*SREBF1* and *ABCG1*
	MHO (n=65)	74 F/26 M	43.08 ± 12.62		VAT (Total)	*ABCG1*, *SREBF1*	No differences in DNA methylation in subjects with MUHO
	MUHO (n=35)						Methylation risk score is significantly different between subjects with MUHO and subjects with MHO (0.4644 *vs* -0.02802)

NW – Subjects that are with normal weight, MHO – Subjects that are metabolically healthy and with overweight/obesity, MUHO - Subjects that are metabolically unhealthy and with overweight/obesity; SAT, Subcutaneous Adipose Tissue; VAT, Visceral Adipose Tissue.

The majority of the DNA methylation epigenome-wide studies relied on an array-based methodology, while 2 studies used the Reduced Representation Bisulfite Sequencing (RRBS) method for identifying DMRs (60, 66). Most studies included adjustments for age, sex and BMI in the analysis. The findings on the genome-wide DNA methylation studies are summarized in **Table 2** and further described in **Supplementary Table 3**.

**Table 2:** Data obtained from epigenome-wide studies assessing DNA methylation in dysglycemic states or correlated with glycemic parameters.

Reference	Population for DNA methylation analysis	Sex distribution of respective groups	Ages of respective groups (years)	Experimental design (studied glycemic parameters)	Tissue	Method	Statistical Adjustments	Main Findings (NW/MHO *vs* MUHO)
Andersen E. et al. (60)				Cross-sectional Case-control (MHO *vs* MUHO)				100 DMRs (53 hypomethylated and 47 hypermethylated in subjects with MUHO *vs* subjects with MHO)
	MHO (n=14) MUHO (n= 14)	MHO=6 F/8 M MUHO=6 F/8 M	MHO=42.1 ± 6.2 MUHO=47.1 ± 3.2		VAT (Pre-adipocytes)	RRBS	FDR 10%	46 genes were differently methylated in over 20% when comparing subjects with MUHO *vs* subjects with MHO
								>20% hypermethylation in subjects with MUHO:
								*C16orf45*, *GMPR*, *ACOX3*, *KDM4B*, *MIEF2*, *TBC1D1*, *GLUD1*, *THUMPD3-AS1*, *PFKFB3*, *IL13RA1*, *B4GALT1*, *FAM120C*, *HDAC2*, *PCF11*, *GGA3*, *LOC339529*, *CIC*, *FAM213B*, *NKRF*, *CORT*, *SMIM1*, *LRRC37A11P* and *GALNT6* were hypermethylated in the promoter region, whereas *STARD13*, *ZFP36L1*, *INIP*, *LINC0059* and *CHN2* were hypermethylated in the intron region and *MORN3* in the distal intragenic region
								>20% hypomethylation in subjects with MUHO:
								*QKI*, *CDO1*, *ARMC3*, *MOB3A*, *PSRC1*, *RAB11FIP2*, *DCAF11*, *LINC01097*, *SH3GL2* and *KCNC3* were hypomethylated in the promoter region, *LOC286238*, *SPAG6*, *RNF223* and *ROBO3* were hypomethylated in distal intragenic, *CAMTA1* and *SUGP1* were hypomethylated in the intron region and *OGG1* hypomethylation in the exon region
								In the MUHO group, *RPL6*, *RNPS1*, *DDX39B* and *HNRNPD* exhibited a positive association between gene expression and methylation and *GTF3C3*, *PRSS12* and *L1TD1* a negative association
Orozco L. D. et al. (66)								After identifying 24 gene candidates, 6 loci were associated with either Matsuda index or HOMA-IR
	METSIM cohort of Finnish male subjects MHO (n=228)	228 M	45-73	Cross-sectional (HOMA-IR, Matsuda, plasma insulin levels, OGGT)	SAT (Total)	RRBS	Bonferroni (p<10^-7^)	Methylation of *AFTPH*/*SLC1A4* was positively correlated with Matsuda index but negatively correlated with HOMA-IR. Methylation of *BOD1*/*CEPB4* and *FASN* were positively correlated with HOMA-IR but negatively correlated with Matsuda Index. *FRMD8*/*SCYL1* and *RAP1GAP2* were negatively correlated with HOMA-IR *LINC01317*/*LINC01320* was positively associated with Matsuda index
								3 (*FASN*, *SLC1A4*, *CPEB4*) of these differentially methylated genes were associated with multiple clinical traits and further explored in the paper
								*FASN*
								Expression is negatively correlated with methylation, BMI and Matsuda index
								*SLC1A4* - upstream
								Methylation was associated with plasma insulin levels, BMI, HOMA-IR and Matsuda index and negatively correlated with gene expression
								Gene expression (qRT-PCR) is positively associated with HOMA-IR
								*CPEB4* - upstream
								Methylation is associated with basal plasma insulin levels, Matsuda index, HOMA-IR
Lee K. et al. (64)	Discovery (A) NW (n=250) MHO (n=200) T2D Cohort (B) NW (n=889) MUHO (n=647)	(A) NW=250 M MHO =200 M (B) NW=442 F/447 M MUHO =283 F/364 M	(A) NW=47.2 ± 5.9 MHO =47.7 ± 5.9 (B) NW=57.76 ± 8.38 MUHO =62.49 ± 8.64	Cross-sectional Case-control ( NW *vs* MUHO)	VAT (Total)	Illumina Infinium Methylation EPIC Kit	Bonferroni (p<1.13*10^-5^)Adjusted for BMI	Promoter methylation in *CISH, PDK4* and *NR4A1* Differential methylation of 3 CpGs was analyzed in relation to T2D, but only *PDK4* showed a significant association

Wang C. *et al.* (70)	Han and Kazak Subjects NW (n=10) MUHO (n=8)	–	–	Cross-sectional Case-control ( NW *vs* MUHO)	VAT (Total)	Illumina Human-Methylation 450K BeadChip	None	In Han subjects with MUHO, 46871 differential methylated sites (DMS) were assayed
								Hypermethylated - 7352 sites (corresponding to 4848 genes) Hypomethylated - 39519 sites (corresponding to 3825 genes)
								In Kazak subjects with MUHO, 22046 DMS were assayed
								hypermethylated - 6812 sites (corresponding to 3825 genes)
								hypomethylated - 15234 sites (no information)
								In gene promoter region, the DMS with reversely expressed genes (RNA microarray) were collected. No common methylation sites were observed in both ethnic groups
								Han subjects with MUHO
								14 hypermethylation sites in promoters corresponding to 12 down-regulated genes
								5 hypomethylation sites in promoters corresponding to 5 up-regulated genes
								Kazak subjects with MUHO
								150 hypermethylation sites in promoters corresponding to 110 down-regulated genes
								52 hypomethylation sites corresponding to 43 up-regulated genes
								*MFSD1, ARHGEF1, ARHGEF1, MYCN* and *SCARB1*
								Upstream regions of these genes are associated with DMS in subjects with MUHO
Crujeiras A. B. et al. (63)	Caucasian subjects (A) Discovery Cohort: MHO (n=5) MUHO (n=7) (B) Validation Cohort: MHO (n=13) MUHO (n=11)	(A) MHO=4 F/1 M MUHO=6 F/1 M (B) MHO=12 F/1 M MUHO=10 F/1 M	(A) MHO=53.2 ± 10.3 MUHO=46.5 ± 9.3 (B) MHO=45.3 ± 11.4 MUHO=48.8 ± 6.8	Cross-sectional Case-control (MHO *vs* MUHO)	VAT (Total)	(A) Illumina Human-Methylation 450K BeadChip (B) Targeted (*ZNF714*)	FDR 5%	982 individual CpG sites (associated with 538 unique genes) that exhibit differential DNA methylation between subjects with MUHO and subjects with MHO
								IR-related DMCpGs were distributed mainly in the intergenic and open-sea regions, being associated with hypermethylation. CpG islands and promoter regions were associated with hypomethylation
								Gene ontology has shown associations with the following pathways: gene (gene region, difference between subjects with MUHO and subjects with MHO)
								Cell adhesion
								Hypermethylated - *COL9A1* (body, 0.06), *COL11A2* (body/body, 0.15/0.05), and *CD44* (body, 0.06)
								Hypomethylated - *MUC4* (body, -0.06) and *ADAM12* (body, -0.06)
								Transcriptional regulation functions
								Hypermethylated - *HOXC4* (5’UTR, 0.05), *TET1* (5’UTR, 0.06), *FOXD2* (TSS1500, 0.34), and *NRBF2* (1st exon/5’UTR, 0.13)
								Hypomethylated - *PER3* (5’UTr/1st exon, -0.06), *GATA4* (body/body, -0.07/-0.07), *PRDM8* (3’UTR,-0.06), *TXNIP1* ((3’UTR,-0.06), and *ZNF714* (TSS200/5’UTR;1st exon/5’UTR/5’UTR/5’UTR, -0.32/-0.23/-0.22/-0.25/-0.37)
								Signal transduction function
								Hypermethylated - *IGF2R* (body/0.06), *CXCL12* (body, 0.05), *CXCL13* (TSS200, 0.09), FGF7(5’UTR, 0.06), and *FGF14* (body, 0.05)
								Hypomethylated - *TNFSF14* (body, 0.05) and *ADCY9* (body/body, -0.06,-0.07)
								Metabolic function - not significant in gene ontology analysis
								Hypermethylated - *HADACM*, *SCG3*
								Hypomethylated - *RAMP1*, *MUC4*, *SULT1B1*, *HOX13*, *TBX5* (4CpG sites), *FAMBA1*
								*ZNF714*
								Hypomethylated in subjects with MUHO (discovery and validation cohorts) - mean delta b=-0.17
								Increased mRNA expression (qRT-PCR)
Rönn T. et al. (69)	(A) Males with MHO (n=96) (B) Females with MHO (n=94) (C) Males with MHO (n=37) Females with MHO (n=67)	(A) 96 M (B) 94 F (C) 67 F/37 M	(A) 32.4 ± 12.8 (B) 29.2 ± 4.2 (C) 52 ± 11	Cross-sectional (HbA1c)	SAT (Total)	Illumina Human-Methylation 450K BeadChip	FDR 5%	Males with MHO discovery cohort
								Average DNA methylation level for all 456 800 CpG sites throughout the genome correlated negatively with HbA1c (statistically significant for 1st exon, 5’UTR, TSS1500 and TSS200)
								DNA methylation of 711 individual CpG sites was significantly associated with HbA1c of which 541 are annotated to 583 unique genes and 170 CpG sites are intergenic. CpGs were mostly associated with gene bodies (25.1%), intergenic regions (19.5%) and TSS1500 (19.2%)
								99 (14%) showed positive and 612 (86%) showed negative correlations between adipose tissue DNA methylation and HbA1c
								The most significant correlation between HbA1c and adipose tissue DNA methylation was seen for a CpG site upstream (TSS1500) of *ANKRD11*
								Females with MHO validation cohort
								Seven CpG sites with DNA methylation significantly associated with HbA1c: two (*CYB5R3*, body; *WSCD2*, TSS1500) with a positive and five (*TNFSF11*, 5’UTR; *GRIK1*/*NCRNA00110*, body/TSS1500; *TNRC18*, body; *MEP1A*, TSS200; *PIGL*, 3’UTR) with a negative coefficient The strongest correlation was observed for a CpG site upstream *TNFSF11*
								None of these seven sites were significantly associated with HbA1c in Males with MHO
								12 overlapping CpG sites in the same direction were found between BMI and HbA1c
								Correlations were performed with DNA methylation associated with HbA1c and mRNA expression (microarray) (56% of the analyzed CpG sites were unique)
								891 positive correlations
								1095 negative correlations
Pietiläinen K. H. et al. (67)	(A) BMI-concordant twin-pairs MHO (n=22) (B) BMI-discordant twin-pairs MHO (n=48) Not able to obtain AT from 4 subjects (Original n=52)	(A) 8 F/14 M (B) 34 F/18 M	(A) 23-36 (B) 23-36	Cross-sectional (glucose, insulin, Matsuda Index and HOMA)	SAT (Total, Isolated adipocytes)	Illumina Human-Methylation 450K BeadChip	FDR 25%	Obesity was not associated with global methylation differences in SAT
								22 differentially methylated CpGs between BMI-discordant co-twins
								*EHBP1L1* (body, 0.15), *ACSF3* (body, -0.04), *BAG6* (promoter), *CHST11* (body), *E2F5* (body) and *ASAP2* (body) - hypomethylated genes
								Methylation is positively correlated with Matsuda Index and negatively correlated with insulin and HOMA-IR
								*AXIN2* (body), *SERPINF1* (3’UTR), *MRPL23* (body), *MAML3* (body), *RBPMS* (body), *SORBS3* (body), *FGFRL1* (body), *CCDC92* (3’UTR), *ZBTB16* (body), *MAD1L1* (body) and *TBC1D16* (body)- hypermethylated genes
								Methylation is negatively associated with Matsuda Index and positively correlated with insulin and HOMA-IR
								RNA microarray analysis (fold change): *CHST11* (0.33), *ZBTB16* (-0.26), *E2F5* (-0.11), *FGFRL1* (-0.36), *ASAP2* (0.17), *EHBP1L1* (0.15), *RBPMS* (-0.29), *TBC1D16* (0.52) and *MRPL23* (0.12) were differentially expressed between BMI-discordant twins. These results were validated by qRT-PCR in another BMI-discordant cohort
Nilsson E. et al. (65)	(A) BMI and T2D discordant MZ twin pairs MHO (n=14) MUHO (n=14) (B) MHO (n=28) MUHO (n= 28)	(A) MHO=5 F/9 M MUHO= 5 F/9 M (B) MHO =13 F/15 M MUHO=13 F/15 M	(A) MHO=67.6 ± 7.7 MUHO= 67.6 ± 7.7 (B) MHO =74.3 ± 4.3 MUHO=74.5 ± 4.2	Cross-sectional Case-control (MHO *vs* MUHO)	SAT (Total)	Illumina Human-Methylation 450K BeadChip	FDR 15%	MZ twins discordant for glycemic status
								No significant differences in average DNA methylation
								Methylation level was higher within the gene body, 3’UTR, and intergenic regions, and lower in TSS1500, TSS200, 59 UTR, and the first exon. Differences were the same irrespective of glycemic status
								Case-control cohort (MHO *vs* MUHO)
								15627 sites associated with 7046 genes were differentially methylated in subjects with MUHO (over-represented in the gene body/enhancer regions and under-represented in TSS1500, TSS200)
								6754 sites were hypermethylated and 8873 sites were hypomethylated
								123 sites presented differential methylation representing 50 T2D candidate genes including *IRS1, PPARG, KCNQ1*, and *TCF7L2* in subjects with MUHO
								127 sites representing 65 candidate genes for obesity were differentially methylated in subjects with MUHO
								~91% of the CpG sites that exhibit differential DNA methylation due to increased BMI or glucose levels in subjects with MHO changed in the same direction as methylation in subjects with MUHO
								*S100A4* and *SLC37A2*
								Both cohorts present hypomethylation of these genes and increased expression in subjects with MUHO (microarrays)
Arner P. et al. (61)	MHO (n=40) MUHO (n=40)	MHO=40 FMUHO=40 F	MHO=35.7 ± 5.7 MUHO=36.4 ± 6.3	Cross-sectional Case-control (IS *vs* IR)	VAT (Total) SAT (Total)	Illumina Human-Methylation 450K BeadChip	FDR 10%Adjusted for BMI and Age	No changes in global DNA methylation profile in SAT or VAT in subjects with MUHO. No DMS were found in SAT or VAT after FDR correction
								Microarrays
								In VAT, merging the 51 differentially associated expressed genes in VAT with the 10,217 DMS (uncorrected for DMRs) identified 18 IR associated genes containing a total of 29 DMS in subjects with MUHO. In VAT, four genes (*CA3, CDKN2C, DAPK2, PAIP2B*) displayed direct correlation between gene expression and methylation
								In SAT, merging the 647 differentially expressed genes in SAT with the 10,746 DMS, 223 IR-associated genes containing a total of 336 DMS were identified in subjects with MUHO. 29 genes (*ABCC3, ADAMTS15, ADAMTS2, ALDH1A1, AMPD3, ATP10A, C1QTNF7, CHST3, COL5A1, CPED1, CYP4X1, EDNRA, GPC1, IRF8, KCNAB1, NECAB1, NIPSNAP3B, PCMTD1, PTGER3, RHOT1, RNF217, ROR1, SAMD4A, SEMA3G, SH3PXD2, SCLC4A4, SYNE2, TSPYL2, VTRNA1-*3) displayed direct correlation between gene expression and methylation
Barajas-Olmos F. et al. (62)	MHO (n=23) MUHO (n=23)	MHO=16 F/7 M MUHO=16 F/7 M	MHO=40.96 ± 6.19 MUHO=41.75 ± 9.93	Cross-sectional Case-control (MHO *vs* MUHO)	VAT (Total) SAT (Total)	Illumina Human-Methylation 27K BeadChip	None	Global average methylation levels between SAT and VAT are highly correlated (0.99)
								VAT
								340 DMCs, including 78 positive and 262 negative in subjects with MUHO
								*LCAT*, *FOXA2*, *KCNQ1* and *GCKR* are genes associated with obesity or T2D
								*FUCA1, C4orf33, PRAP1, SNX4, TMEM109, ZNF597, SLC9A2* were overlapped with other tissues
								SAT
								68 DMCs, including 29 positive and 39 negative in subjects with MUHO
								*IRS1*, *LEP* and *ADIPOQ* are genes associated with obesity or T2D
								*MIA2, PSMD5*, *PAMR1*, and *SUMO3* were overlapped in other tissues
								DMCs in *CCDC185, MTHFD2*, and *SUMF1* were overlapped between SAT and VAT
								RNA microarray - genes with the highest altered DNA methylation (>|5%|) and altered expression in subjects with MUHO
								SAT - *DST, MGAT4C, LEP* and *ZNF3*
								VAT - *BRDT, C14orf105, EDNRB, HMP19, PSG6* and *SNX4*
								Most of these genes have not been previously related with T2D
Ribel-Madsen R. et al. (68)	Danish T2D discordant MZ twin pairs MHO (n=5) MUHO (n=5) SAT not available in 7 pairs (Original n in each group=12)	MHO=6 F/6 M MUHO=6 F/6 M	MHO=40.96 ± 6.19 MUHO=41.75 ± 9.93	Cross-sectional Case-control (MHO *vs* MUHO)	SAT (Total)	Illumina Human-Methylation 27K BeadChip	Corrected for multiple testing (Westfall-Young resampling method)	Overall methylation did not differ according to metabolic status
								In a gene candidate approach (136 sites)
								Promoters of *CDKN2A* (12.6 ± 1.9% *vs* 16.6 ± 2.1%) and *HNF4A* (75.2 ± 3.8% *vs* 70.5 ± 3.7%)) were differentially methylated in subjects with MUHO after correction for multiple testing
								In a explorative approach (26850 sites)
								Promoters of *ZNF668* (3.4 ± 0.5% *vs* 2.6 ± 0.5%), *HSPA2* (12.1 ± 1.6% *vs* 14.8 ± 1.6%), *C8ORF31* (35.1 ± 6.0% *vs* 26.9 ± 5.4%), *CD320* (1.4% ± 0.4% *vs* 1.9 ± 0.3%), *SFT2D3* (67.9 ± 3.7% *vs* 74.5 ± 3.6%), *TWIST1* (10.6 ± 0.8% *vs* 12.6 ± 0.8%), *MYO5A* (2.6 ± 0.4% *vs* 3.4 ± 0.5%), were differentially methylated in subjects with MUHO after correction for multiple testing

NW – Subjects that are with normal weight, MHO – Subjects that are metabolically healthy and with overweight/obesity, MUHO - Subjects that are metabolically unhealthy and with overweight/obesity; SAT, Subcutaneous Adipose Tissue; VAT, Visceral Adipose Tissue.

After completing the data analysis of this systematic review on AT, an additional non-systematic bibliographical search for EWAS conducted in WB of patients with MHO and MUHO was performed in order to understand whether common methylated CpG sites could be identified in AT and WB.

Our search strategy found two papers focused on the association between AT histone modifications and T2D or altered glycemic traits (71, 72). Both papers reported cross-sectional studies with a case control approach. The main findings of the histone studies are presented in **Table 3**. Finally, from the 23 papers identified as meeting the criteria for inclusion in this systematic review, two papers were found to describe changes in lncRNA expression in dysglycemic states in subjects with obesity (73, 74). **Table 4** describes these main findings and possible targets identified in these studies.

**Table 3:** Data obtained from studies assessing histone modifications in dysglycemic states or when correlated with glycemic parameters.

Reference	Population for histone analysis	Sex distribution of respective groups	Ages of respective groups (years)	Experimental design (studied glycemic parameters)	Tissue	Method	Main Findings (NW/MHO *vs* MUHO)
Jufvas A. et al. (72)	NW (n=14)	NW=14 F	NW=64.4 ± 8.7	Cross-sectional Case-control	SAT (Isolated adipocytes)	SDS-PAGE and Immunoblotting	Subjects with MUHO present 40% higher H3K4me3 than L subjects or subjects with MHO
	MHO (n=19)	MHO=19 F	MHO=60.2 ± 11.4	(NW/MHO *vs* MUHO)			Subjects with MHO present 37% lower H3K4me2 than L subjects
	MUHO (n=10)	MUHO =8 F/2 M	MUHO =55.2 ± 15.2				No significant differences between groups for H3K9me2
Castellano-Castillo D. et al. (71)	NW (n=10)	NW=6 F/4 M	NW=54.40 ± 13.93	Cross-sectional Case-control	VAT (Total)	Chromatin immunoprecipitation assay	Increased H3K4me3 in *E2F1*, *LPL*, *SREBF2*, *SCD*, *PPARG* and *IL6* promoters in subjects with MUHO *vs* L subjects
	MHO (n=10)	MHO=7 F/3 M	MHO=40.50 ± 8.34	(NW/MHO *vs* MUHO)			Positive correlation between H3K4me3 in gene promoters and HOMA-IR, glucose and insulin for *E2F1, LPL, SREBF2, SCD, PPARG* and *IL6*
	MUHO (n=9)	MUHO=6 F/3 M	MUHO=47.11 ± 8.28				Positive correlation between H3K4me3 in *TNF* gene promoter and glucose
							Decreased mRNA expression of *LPL, SCD* and *PPARG* in subjects with MUHO *vs* L subjects
							Increased mRNA expression of *TNF* and *IL6* in subjects with MUHO *vs* L subjects
							Positive correlation between gene expression and insulin and HOMA-IR for *LEP, IL6* and *TNF. IL6* expression was also positively correlated with glucose
							Negative correlation between gene expression and insulin and HOMA-IR for *SREBF2* and *SCD*

NW – Subjects that are with normal weight, MHO – Subjects that are metabolically healthy and with overweight/obesity, MUHO - Subjects that are metabolically unhealthy and with overweight/obesity; SAT, Subcutaneous Adipose Tissue; VAT, Visceral Adipose Tissue.

**Table 4:** Data obtained from studies assessing lncRNA expression changes in dysglycemic states or when correlated with glycemic parameters.

Reference	Population for lncRNA analysis	Sex distribution of respective groups	Ages of respective groups (years)	Experimental design (studied glycemic parameters)	Tissue	Range of analysis for AT samples	Main findings	Predicted/tested targets of the registered lncRNAs(MHO *vs* MUHO)
Gao H. et al. (73)	MHO (n=40)	MHO=40 F	MHO=36 ± 6	Cross-sectional	VAT (total)	Genome Wide	Two cohorts were analyzed in this article: the first checking non-obesity *vs* obesity; the second subjects with MHO *vs* subjects with MUHO being based on a previous work from Arner et al. (61). This report will be based on the results from the second cohort	*CATG00000111229.1* *In silico* analyses and data from stimulated and non-stimulated lipogenesis/lipolysis have shown that this lncRNA affects inflammation pathways *In vitro* analyses have shown that this lncRNA is positively correlated with *PPARG, INSR, KLF15, SREBF1, IGF1R, PPARA, MAPK1, PPARD, SREBF2, SCAP, ATP7B, THRB* and *FASN* pathways; and that is negatively correlated with *MAP4K4, SYVN1, TFGFB1, ACOX1, STAT3, INSIG1, STAT1, POR* and *INSIG2* signalling pathways
	MUHO (n=40)	MUHO=40 F	MUHO=36 ± 6	Case-control	SAT (total)		No expression differences were found in VAT samples (FDR<0.05)		
				(MHO *vs* MUHO)			44 lncRNAs in intergenic regions were found to be differentially expressed in SAT samples in subjects with MUHO, but only 16 were manually curated for posterior analysis (ROC plots)		
							*CATG00000087873.1, CATG00000106343.1, CATG00000111229.1, ENSG00000229961.1, ENSG00000249378.1, ENSG00000256551.1*	*ENSG00000235609.4*
							Expression is increased in SAT, in subjects with MUHO (average AUC range: 70.5% to 76.6%)	*In silico* analyses and data from stimulated and non-stimulated lipogenesis/lipolysis have shown that this lncRNA affects lipid metabolism pathways
							*CATG00000000027.1, CATG00000085516.1, ENSG00000226891.2, ENSG00000229108.1, ENSG00000235437.3, ENSG00000235609.4, ENSG00000236849.1, ENSG00000250237.1, ENSG00000253434.1, ENSG00000259820.1*	*In vitro* analyses have shown that this lncRNA is positively correlated with *PPARG, INSR, KLF15, IGF1R, PPARA, PPARD, PPARGC1A, FAS* and *TFAM* pathways; and that is negatively correlated with *MAP4K4, SYVN1, TFGFB1, ACOX1, STAT3, KDM5A* and *INS* signalling pathways
							Expression is decreased in SAT, in subjects with MUHO (average AUC range: 70.7% to 78.3%)	It was shown that approximately 500 genes are affected by the former lncRNAs, with 10% being shared in between. Both are positively correlated with *PPARG, INSR, KLF15, PPARA* and *PPARD* signalling pathways and negatively correlated with *MAP4K4, SYVN1, TGFB1* and *ACOX1*. However, some of the genes associated with these pathways were differentially associated with each lncRNA. Inhibition of both was associated with decreased lipolysis, adipogenesis and release of adiponectin
							A crosscheck and validation with real-time PCR were made with data from the first cohort, where 3 lncRNAs expression patterns were determined	*CATG00000000027.1*
							*ENSG00000235609.4*, *CATG00000000027.1*	*In silico* analyses and data from stimulated and non-stimulated lipogenesis/lipolysis have shown that this lncRNA affects lipid metabolism pathways
							Expression is decreased in SAT, in subjects with MUHO (p<0.01)	
							*CATG00000111229.1*	
							Expression is increased in SAT, in subjects with MUHO (p<0.001)	
Shi Y. et al. (74)	NW/MHO (n=6)	–	–	Cross-sectional Case-control (MHO *vs* MUHO)	VAT (Isolated adipocytes)	Targeted (*GAS5*)	*GAS5* Expression is decreased in subjects with MUHO in both samples (p<0.0001)	*In vitro* analyses have shown that *GAS5* modulates positively the insulin pathway in human adipocytes and preadipocytes by serving as a riboactivator in the *INSR* promoter (first assessed through an *in silico* analysis)
	NW/MUHO (n=6)				SAT (Isolated adipocytes)			

NW – Subjects that are with normal weight, MHO – Subjects that are metabolically healthy and with overweight/obesity, MUHO - Subjects that are metabolically unhealthy and with overweight/obesity; SAT, Subcutaneous Adipose Tissue; VAT, Visceral Adipose Tissue.

### DMR Calls in Illumina 450K Arrays

DMRs between cases and controls were identified from the DNA methylation studies that used the Infinium HumanMethylation450 BeadChips (Illumina) platform. DMRs were defined as gain or loss of methylation above the cut-off of 5%.

For the purpose of establishing the genomic distribution of the DMRs, a single researcher (IS) used the HumanMethylation450 v1.2 Manifest File (https://emea.support.illumina.com/downloads/infinium_humanmethylation450_product_files.html) to sort the Illumina 450K CpGs according to their UCSC RefGene_Group category, i.e. TSS1500, TSS200, 1st Exon, 5’UTR, Body or 3’UTR. We identified a total of 297,815 and 1,996 CpG in the 450K array and our data set, respectively, that associated a unique UCSCRefGene group category. IS then applied χ^2^ tests with Yates’ correction to each of these categories.

### Functional Annotation and Enrichment Analysis

DAVID (Database for Annotation, Visualization and Integrated Discovery; v6.8 http://david.abcc.ncifcrf.gov/, accessed March 2021) was performed by IS to assess whether there was a significant enrichment for particular biological processes, or molecular functions, within the gene lists that associated DMRs in AT of individuals with MHO or MUHO (n = 2,139 genes). Enriched gene ontology (GO) terms with a FDR < 0.05 were considered significant. These terms were then clustered semantically using REViGO (Reduce and Visualize GO) (75), which removes redundancy. IPA (Ingenuity Pathway Analysis) was used by IS to identify networks significantly enriched in genes that associated DMR changes between AT of individuals with MHO or MUHO, as well as to predict significantly enriched upstream regulators (i.e. Transcription Factors (TFs)) for our set of genes. To search for enrichment of TF binding sites at DMRs, we first retrieved the 122 bp DNA sequences (60 bp on each side of the CpG) from the HumanMethylation450 v1.2 Manifest File. These sequences were then analyzed using AME (Analysis of Motif Enrichment v4.12.0 – http://meme-suite.org/tools/ame) by selecting *Homo sapiens* and HOCOMOCO Human (v11 FULL) as motif database. We then selected the average odds score as the scoring method and Fisher’s exact test. Network visualization was performed using IPA.

## Results

### DNA Methylation

The DNA methylation studies included in this systematic review included either targeted, i.e. hypothesis-driven studies, or used unbiased methylome-wide approaches.

#### Targeted DNA Methylation Studies

Targeted epigenetic studies evaluated AT DNA methylation levels of several candidate genes, either by comparing methylation profiles of individuals with MUHO to controls, or by performing correlation analyses between levels of DNA methylation and glycemic/IR parameters (**Table 1**). Altogether, these studies profiled DNA methylation for 14 genes with established roles in AT and/or glycemic control, ranging from adipokines (*TNFA, ADIPOQ*) to a glucose transporter (*SLC2A4*). Of these 14 genes (**Table 1**), *ADIPOQ* and *TNFA* had gene methylation frequency altered in MUHO (59), whilst *FGF21* (58), *INSR, SLC2A4* (56) and *FKBP5* (57) were associated with DMRs that correlated negatively with gene expression. Meanwhile, *MCP1* (59), *HIF3A* (55), *LEP* (53), *C3* (52), *SREBF*, *ABCG1* (54), *PIK3R1* (56) and *GR* (57) showed no differences between subjects with MUHO and NW/MHO controls, or showed no correlation with glycemic parameters. Association between DNA methylation with fasting glucose were found for *ADIPOQ* (positive association) and *TNFA* (negative association), although there is some contradicting information regarding *ADIPOQ* since another study found no relationship between *ADIPOQ* and any glycemic parameters (53). HOMA-IR showed a positive correlation with methylation at *INSR*, *SLC2A4* (56) and *FKBP5* (57).

#### Genome-Wide DNA Methylation Studies

Most genome-wide studies included in this review sought to evaluate DNA methylation profiles in the AT of subjects with MUHO in comparison to a control NW/MHO group (case-control studies) or attempted correlations between DNA methylation changes and glycemic/IR parameters (summarized in **Table 2** and further described in **Supplementary Table 3**).

##### Correlation Between DNA Methylation With Glycemic/IR Parameters

DNA methylation at specific loci in AT was reported to be correlated with HbA1c levels with a strong sex bias. In men, DNA methylation of 583 unique genes was significantly correlated with HbA1c (69), with the vast majority showing negative correlations. In contrast, in an all-female cohort, only 7 unique genes were significantly associated with HbA1c (69). In 2 of those genes (*CYB5R3* and *WSCD2*), HbA1c was positively correlated with DNA methylation, meanwhile the other 5 (*TNFSF11*, *GRIK1/NCRNA00110*, *TNRC18, MEP1A* and *PIGL*) showed a negative correlation (69) (**Table 2**).

In other studies, HOMA-IR (66, 67) was associated with DNA methylation. *EHBP1L1*, *ACSF3*, *BAG6*, *CHST11*, *E2F5*, *ASAP2*, *BOD1*/*CPEB4* and *FASN* all showed a positive correlation between HOMA-IR and DNA methylation, whereas *AXIN2, SERPINF1, MRPL23, MAML3, RBPMS, SORBS3, FGFRL1, CCDC92, ZBTB16, MAD1L1, TBC1D16, AFTPH/SLC1A4, FRMD8/SCYL1* and *RAP1GAP2* demonstrated a negative correlation (**Table 2**).

As for correlations between DNA methylation and the Matsuda Index, *AXIN2*, *SERPINF1*, *MRPL23*, *MAML3*, *RBPMS*, *CCDC92, LINC01317*/*LINC01320, AFTPH*/*SLC1A4* showed a positive correlation and *EHBP1L1*, *ACSF3*, *BAG6, BOD1*/*CPEB4 and FASN* showed a negative correlation (66, 67) (**Table 2**).

##### DNA Methylation Signatures in Subjects With MUHO vs Subjects With MHO

An interesting RRBS-based study used preadipocytes derived from MUHO cases compared to MHO cases and found evidence for 100 DMRs, with almost half (n=44) of those DMRs showing significant changes >20% (**Table 2**). Interestingly, the list of hypermethylated genes in the group with MUHO >20% features two major ‘erasers’ of histone modifications (*KDM4B*, codifying a lysine demethylase and *HDAC2*, codifying a histone deacetylase).

Results from case-control EWAS Illumina-array based studies yielded very heterogeneous findings, likely reflecting the sex, fat depot and age-related differences between cohorts (**Table 2** and **Supplementary Table 3**). In total, several thousand DMRs were identified in the AT of individuals that had MUHO, although without significant overlap between the individual studies (60–65, 68, 70).

We then compiled all the data from the relevant studies (60, 62, 63, 65, 68), irrespective of magnitude of DNA methylation differences, and found statistically significant DMRs at 5648 CpGs, with a global mean change in methylation of only -0.19% for MUHO when compared to MHO (**Supplementary Table 3**). This finding suggests that DNA methylation differences are subtle and localized to many loci, as it might be expected for common diseases.

Small changes in DNA methylation raise the question of how impactful they might be in terms of gene expression and cellular function. However, small DNA methylation differences across common gene pathways may have a cumulative effect. For a more focused analysis we used 5%, as lower cutoff limit, for DNA methylation differences, which is an approach followed by two of the studies reviewed here (62, 63). We identified 2494 DMRs with differences greater than 5%, of which 204 were greater than 10% and 78 greater than 20% between subjects with MUHO and their corresponding controls. These 2494 DMRs were mapped to 2136 unique genes, of which 983 associated gain of methylation and 1,153 loss of methylation in the groups with MUHO in relation to their respective controls (**Supplementary Table 4**). Most of the DMRs with differences greater than 5% were found using the Illumina 450K arrays and 1,996 could be assigned to a specific genomic feature.

Importantly, we observed that among the AT-DMRs genes, some were not previously strongly or unequivocally linked to adipogenesis nor dysglycemia, so we decided to focus on the ten most differentially methylated ones. Among these genes, 3 (*FCGBP, KCNC3* and *ARMC3*) were hypomethylated and 7 (*HLA-DRB6*, *SMIM1*, *CAPN8, INIP*, *NKRF, GALNT6* and *STARD13*) were hypermethylated in MUHO (for further information see **Supplementary Table 3**, with the 10 genes highlighted with an asterisk**)**. Next, these potentially novel genes are described in more detail.

The greatest methylation differences were identified in genes that belong to the human leukocyte antigen (HLA) system, with 3 of the most differentially methylated CpGs belonging to *HLA-DBR6*, *HLA-DBR1* and *HLA-DQA1*, all found to be hypermethylated in MUHO. *HLA-DRB6* is associated with immunological and neurological disorders such as multiple sclerosis (76), Alzheimer’s disease (77) or chronic pain (78). Nevertheless, given that the HLA system regulates the adaptive immune response, it can be hypothesized that this gene may play a role in mediating the AT inflammatory state in MUHO.

*SMIM1* was found to be hypermethylated in MUHO. This gene codifies a protein that is involved in red blood cell formation being also an antigen in the Vel blood group (79). This blood group is more prevalent in individuals of Nordic ancestry and, so far, there is no known connection between this protein and dysglycemia. It may be overrepresented in our study, as the study that reported this DMR was performed in a Danish population (60).

*STARD13* was another gene found to be hypermethylated in MUHO. *STARD13* encodes the protein StAR-related lipid transfer protein 13, a Rho GTPase-activating protein closely associated with hepatic cancer (80). Besides being linked with actin cytoskeletal organization, this protein also seems to influence other biological activities (81), such as insulin secretion (82), and induction of mitochondrial phosphatidylglycerolphosphate synthase activation in response to ceramides (83). This may be particularly relevant in the adipocyte’s metabolic deregulation, as ceramides play an important role in glucose uptake and metabolism (84). *ARMC1* and *ARMC3* are both hypomethylated in MUHO. ARM domain-containing proteins, such as ARMC1 and ARMC3, function in signal transduction, development, cell adhesion and mobility, and tumor initiation and metastasis (85). In signal transduction, this protein family is closely linked to Wnt/beta-catenin signaling pathway (85), involved in insulin sensitivity (86) and adipocyte differentiation (87). The gene that encodes the voltage-gated potassium channel protein KV3.3, *KCNC3*, is hypomethylated in MUHO. This gene codifies a type of potassium channel that is mainly linked to spinocerebellar ataxia 13 (88), but it also seems to play a role in adipocyte differentiation from bone marrow-derived human mesenchymal stem cells (89). Furthermore, there may be a link between this protein and TANK-binding kinase 1 (90), a protein that seems to be important in AT metabolic regulation (91). *FCGBP*, the gene that codifies the Fc fragment of IgG binding protein, which binds the Fc portion of IgG molecules, is hypomethylated in MUHO. This protein is expressed in mucin secreting cells in tissues such as the colon, small intestine or gall bladder (92). It is hypothesized to be involved in anti-inflammatory processes (93), and if this role is confirmed in the AT, it may confer some protection against the obesity induced low-grade inflammatory state (94). Furthermore, higher levels of FCGBP are positively correlated with M2 response, further supporting the potential role in determining immune cell infiltration phenotype (95). *GALNT6* encodes the polypeptide N-acetylgalactosaminyltransferase 6 (GalNAc-T6), a protein highly associated with several types of cancer and mostly absent in healthy tissues (96, 97). GalNAc-T6 has several identified targets, two of which that are related with inflammation, CD44 and CD74, with the last being a specific target of this protein (96). Both CDs (98) have been linked with AT inflammation with more preponderance for CD44 (99). *NKRF*, the gene that expresses the NF-kB-repressing factor, is hypermethylated in MUHO. This protein directly represses NF-kB (100), an important player in the inflammatory signaling that mediates the response to TNF-α, thus being involved in insulin resistance (101, 102). Furthermore, NF-kB is vital to TNF-α-induced lipolysis in adipocytes (103). Lastly, NF-kB expression and activity change during adipocyte differentiation (104). Finally, there are two genes that present large methylation changes whose functions are either unknown or unrelated to the adipocyte. These are *INIP* and *CAPN8*, both found to be hypermethylated in MUHO. INIP is involved in DNA repair, but its precise biological functions are unknown (105). As for CAPN8, it belongs to the mammalian calpain protease family (106), with a specific role in the gastrointestinal tract, where it forms a heterodimer with CAPN9, which seems to be involved in gastric protection (107, 108).

##### In Silico Bioinformatic Analyses Across Studies With DMRs >5% in Subjects With MUHO *vs* Subjects With MHO

**Figure 2:**
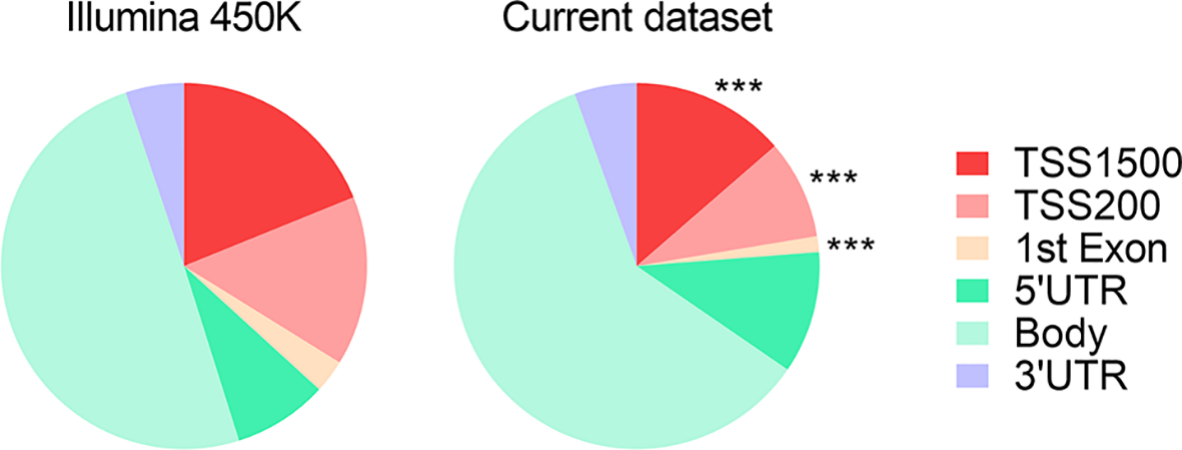
Pie chart representing the proportions of genomic features with associated DMRs between subjects with MUHO and respective controls with MHO. *** indicates *P* < 0.0001 by χ^2^ tests with Yates’ correction. Data derived from refs (63, 65).

We observed that DMRs between subjects with MUHO and their respective controls, irrespective of their direction of change, tended to have a skewed distribution, with a significant depletion around the promoter regions (**Figure 2**).

**Figure 3:**
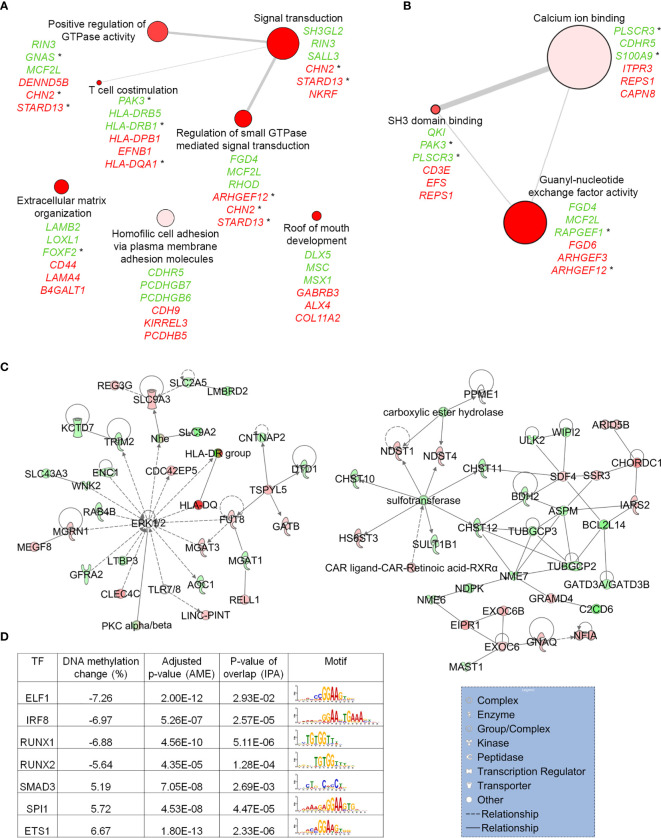
*In silico* enrichment analyses of genes that associate DNA methylation changes between subjects with MUHO, compared to controls with MHO. Top scoring biological processes **(A)** and molecular functions **(B)** enriched in genes that associate DNA methylation changes above the cut-off of 5%. For each GO term, top three genes with the highest levels of DNA methylation changes are shown (loss of DNA methylation in green and gain of DNA methylation in red). Genes with an associated * have been previously been connected with regulation of glucose homeostasis (see **Supplementary Table 5**). The size of each bubble indicates the frequency of the GO term in the GOA (Gene Ontology Annotation) database. Lines connecting GO terms indicate relatedness and the line width indicates the degree of similarity. **(C)** IPA networks significantly enriched in genes implicated in carbohydrate metabolism (see also **Supplementary Table 6**). Green and red depict genes that associate DNA methylation loss and gain, respectively, in individuals with MUHO. Solid lines represent direct interactions between the two gene products and dotted lines mean that there is an indirect interaction. The legend shown under the second network indicates the type of protein encoded by each gene. **(D)** TFs that associate DNA methylation changes in individuals with MUHO and with significant enrichment of binding sites at genes harbouring differentially methylated CpGs, as determined by AME and IPA analyses. Data derived from studies (60, 62, 63, 65, 68).

Next, in order to understand the biological meaning of the set of genes that associated DNA methylation changes in subjects with MUHO, we applied DAVID functional annotation, followed by REViGO clustering (see Methods and **Supplementary Table 4**). Using this approach, we found significant enrichment of several GO terms, including signal transduction, positive regulation of GTPase activity and calcium ion binding (**Figures 3A, B** **)**, suggesting a potential role for altered intracellular signaling in AT in the pathogeny of obesity-related IR/T2D. Further supporting this potential link, we found that several genes belonging to these GO terms and associating larger DNA methylation differences between subjects with MUHO and their respective controls, have been previously linked with traits such as IR, glucose intolerance and dyslipidemia in rodents, or have been associated with T2D in humans through genetic studies (**Figures 3A, B** and **Supplementary Table 5**).

To understand further the potential roles of the genes that associate DNA methylation changes in subjects with MUHO, we then used IPA and performed network analysis (see Methods). We identified 25 networks enriched in our gene set (**Supplementary Table 6**), two of which are implicated in the carbohydrate metabolism (**Figure 3C**). Importantly, in one of these networks, ERK1/2 is the main central node, a pathway which has been implicated in the development of IR associated with obesity and T2D (109–111). The second network has several more discrete nodes highlighting intracellular trafficking, such as EXOC6, which has been implicated in the GLUT4 translocation in response to insulin signaling (112) and SDF4/Cab45 that has a central role in sorting specific cargo molecules at the trans-Golgi network (113).

We then searched for upstream regulators that might be implicated in the occurrence of DNA methylation changes in AT samples of subjects with MUHO. To this aim, we used two computational approaches. First, we used AME and identified 187 TF motifs significantly enriched within sequences of DNA centered on DMRs (see Methods and **Supplementary Table 7**). Using IPA, we then identified 230 TFs significantly enriched as upstream regulators of genes that associate DMRs in AT samples of subjects with MUHO or MHO (see Methods and **Supplementary Table 4**). Forty-seven TFs were common between the two computational approaches and seven of these associated DMRs (**Figure 3D**). These seven TFs are predicted to regulate 149 of the genes in our dataset (**Supplementary Figure 1**) and include ETS1 (114, 115) SPI1/Pu.1 (116, 117) and SMAD3 (118), which were previously linked with glucose homeostasis.

Altogether, our bioinformatics analyses suggest an important role for altered intracellular signaling as a key event in the development of IR/dysglycemia in some individuals with overweight/obesity and highlight the potential implication of a network of TFs in the pathogeny of obesity-associated T2D.

##### Correlation Between DNA Methylation in AT and in WB

To identify the extent to which the DMRs are specific to AT, on a second stage we then performed a non-systematic search in PubMed for papers describing DNA methylation in WB of subjects with MUHO (39, 40, 46, 61, 62, 69, 119–128). 27 genes associating methylation changes above 5% in AT were also found to carry DMRs in WB, with 8 common CpGs, each located in a single gene, showing concordant methylation patterns in WB and AT: hypermethylation in subjects with MUHO at cg14642338 (*PAMR1*), cg09419670 (*PSMD5*), cg21053323 (*SUMO3*) and cg17878506 (*TBC1D4*); hypomethylation at cg02707176 (*PCDHGA1/PCDHGA4*), cg20050113 (*SLC9A2*), cg19693031 (*TXNIP*) and cg00117018 (*ZNF251*), with an overall correlation coefficient R^2^ = 0.88 (**Supplementary Table 8**).

Interestingly, five of these genes have not been previously linked to dysglycemia and will be discussed in more detail (**Supplementary Table 8**). *PCDHGA1/PCDHGA4* are genes from a cluster that codifies protocadherin gamma proteins. These cell-adhesion molecules are typically associated with brain neuron synapses, but no association has been found with AT biology or glucose sensitivity (129, 130). *PSMD5* codifies the proteasome 26S subunit S5B. Since MUHO is commonly associated with a low-grade chronic inflammation and dysregulation of cellular proteostasis, this gene could potentiate the MUHO phenotype by its role in proteasome 26 assembly. *SLC9A2* codifies a sodium ion-proton exchanger. This transporter has mostly been associated with the gastrointestinal tract and the kidney, regulating cellular pH and volume, with data suggesting that it is mostly localized in the apical epithelial membrane (131, 132). This transporter might be involved in the transport of short chain fatty acids (133), which are speculated to modulate glucose metabolism in humans (134). Also, its role in renal physiology could also intervene in other diabetes comorbidities, such as nephropathy. *SUMO3* codifies a protein of the small ubiquitin-related modifier family, thoroughly described in post-transcriptional protein modification, especially in the context of various diseases where T2D is included (135). SUMO3 was associated with the modification and translocation of ATF5, a transcription factor that has also been previously associated with ß-cell survival (136), from the centrosome, promoting the cell cycle progression (137). *ZNF251* codifies a protein of the zinc finger family. Zinc finger proteins, while being commonly associated with DNA recognition and interaction, are currently known to interact with a plethora of biomolecules, therefore possessing a wide range of functions (138). However, the role of *ZNF251* in human physiology is currently unknown. Even so, this gene has been associated to the promotion of lung cancer through activation of the ERK signalling pathway (139). This signalling pathway is closely associated with AT biology and glucose sensitivity, so one may posit that this gene could affect the MUHO phenotype by the ERK signalling pathway (109).

### Histone Modifications and Long Non-Coding RNAs

Histone modifications have important effects in several biological processes associated with gene expression, DNA replication and chromatin compaction, among others. Methylation of specific lysine residues, such as H3K4, are generally associated with active transcription, while a transcriptional repression is observed when the methylations occurs at H3K9 (140). Although the number of studies performed so far comparing AT of subjects with MUHO or MHO is small (**Table 3**), they demonstrated differences in levels of specific histone modifications, both in terms of global levels (72), as well as at the promoters of key genes implicated in adipogenesis, lipid metabolism and inflammation (71). Additionally, levels of H3K4me3 at specific promoters correlated with HOMA-IR, glucose or insulin levels (71) (**Table 3**).

LncRNAs are known to play important roles in modulating the cells’ transcriptional landscapes, for example by recruiting other epigenetic marks (141). Both targeted (74) and genome-wide approaches (73) compared the expression of lncRNAs in the AT of individuals with MUHO to that of individuals with MHO, which provided proof-of-principle for altered lncRNA expression, particularly in SAT (**Table 4**). Although the number of differentially expressed lncRNAs identified so far is small, some of these, such as *GAS5* (74), *ENSG00000235609.4* and *CATG00000111229.1* (73) have been functionally validated and demonstrated to play specific roles in processes such as regulation of *INSR* gene expression (*GAS5*), adipocyte differentiation and adiponectin release (*ENSG00000235609.4* and *CATG00000111229.1*) (**Table 4**).

## Discussion

In this review, we systematically appraised and discussed the published epigenetic changes identified within the AT, namely DNA methylation, histone modifications and lncRNAs, associated with obesity-related IR/dysglycemic traits and T2D. Our integrative approach, based on bioinformatic analyses, highlighted a number of additional avenues for future research, such as new signaling pathways, novel TFs and blood-based DNA methylation biomarkers that can discriminate between MHO and MUHO individuals.

Hypothesis-driven DNA methylation studies on selected genes provided some good, albeit limited, links between dysglycemia and levels of DNA methylation. Across the reviewed studies, three genes (*FGF21*, *INSR* and *SLC2A4*) associated hypermethylation around the promoter region in subjects with MUHO versus subjects with MHO. DNA methylation at the promoter regions of these genes showed a negative correlation with expression of the corresponding genes (56, 58). Importantly, these targeted studies found significant correlations between glycemic parameters (such as fasting glucose or HbA1c), insulin resistance measurements (such as HOMA-IR) or insulin sensitivity measurements (such as the Matsuda index or QUICKI) with levels of DNA methylation for *ADIPOQ*, *TNFA*, *FKBP5*, *INSR* and *SLC2A4* (56, 57, 59). These comparisons draw some parallels between glucose levels and DNA gene methylation changes in several tissue types from *in vitro* studies (142, 143). Along those lines, all five genes are related to the cellular responses to insulin: *INSR* encodes the insulin receptor (56), *ADIPOQ* encodes an insulin sensitizer (144), *TNFA* encodes a cytokine linked to insulin resistance and was found to be elevated in the serum of subjects with T2D (145), *SLC2A4* encodes GLUT4 that is the major glucose transporter in adipocytes (146) and *FGF21* encodes a hormone-like protein with insulin sensitizing actions in adipocytes (58).

Most EWAS in the context of obesity performed so far focused on assessing DNA methylation levels in WB. These DNA methylation studies have uncovered several DMRs located in or near genes known to be implicated in body weight regulation or glucose homeostasis, which presented significant correlations with BMI (147). Interestingly, data suggests that these DNA methylation changes associated with BMI are a consequence rather than the cause of obesity (39). In addition, a methylation risk score calculated across loci associated with BMI, was found to be strongly predictive for future T2D risk, while methylation patterns observed in peripheral blood cells and other tissues suggest that altered ‘systemic’ methylation is a signature of T2D (148). Furthermore, a large number of these epigenetic signatures tend to occur in genomic regions where genes that are involved in processes related to glucose homeostasis, such as insulin signalling or glucose/lipid metabolism, are located (149). Our analyses also led to the identification of eight differentially methylated CpGs, associated to eight genes, which have tightly correlated changes between MUHO and MHO individuals in WB and AT. Three of these genes – *TBC1D4*, *TXNIP* and *PAMR1* – were previously implicated in dysglycemia (150–152). However, the remaining five genes (*PCDHGA1/PCDHGA4*, *PSMD5*, *SLC9A2*, *SUMO3* and *ZNF251*) to the best of our knowledge, have not been mechanistically associated with dysglycemia. We speculate that these genes could highlight new molecular pathways relevant for AT dysfunction and progression to dysglycemia. Additionally, since these DMRs are strongly correlated between AT and WB, it is possible to envisage that these could be potentially useful biomarkers when considering to develop a CpGs panel to predict the risk of future T2D in MHO.

We, like others, reasoned that investigating epigenetic signatures in peripheral tissues that are metabolically relevant for the pathogenesis of obesity-related metabolic complications, and specifically in the AT, could provide further insights into the role of epigenetic modifications for the mechanisms of the disease (153). Indeed, the limited number of studies performed so far, and described here, highlight distinctive epigenetic signatures in AT of individuals with MUHO compared to AT of individuals with MHO. In contrast to the limited scope of targeted studies, EWAS studies that compare groups of individuals with MUHO and a control population tend to paint us a fuller picture of the epigenetic landscape. Therefore, our analytic approach was to focus on collating all the data, mostly from the EWAS studies reported to date, with the aim of generating potentially meaningful data, in spite of issues related to the high variability between studies. Indeed, despite the heterogeneous nature of the studies reviewed, particularly in terms of selection criteria of the cohorts and tissue types (total SAT/VAT or isolated adipocytes from SAT/VAT, or pre-adipocytes derived from VAT), we catalogued over two thousand DMRs at the cutoff 5%. Interestingly, the majority of the strongest DMRs (>30%) were located at genes that have not been previously linked with adipogenesis or dysglycemia. Among these, three DMRs were located at genes encoding components of the human leukocyte antigen (HLA) system, classically associated with the risk for type 1 diabetes (154), such as *HLA-DBR1* and *HLA-DQA1*, both hypermethylated in MUHO. Other genes that associate strongly hypermethylated DMRs in MUHO include HLA-DBR6, *STARD13*, *GALNT6*, *NKRF*, *INIP* and *CAPN8*. Genes that associate strongly hypomethylated DMRs in MUHO include *ARMC3*, *MOB3A*, *KCNC3* and *FCGBP*. These genes are implicated in a wide range of activities, from ion transport, to signal transduction and transcriptional regulation (86, 90, 100, 104). Their study in the context of AT biology may provide new clues about the mechanisms leading to MUHO.

Although each individual DMR is unlikely to have a big influence on gene expression, particularly those that associate smaller DNA methylation differences, it is possible that their cumulative effects could have a significant impact on progression to dysglycemia (34). Since most of the DMRs catalogued in our review were identified using the Infinium HumanMethylation450 BeadChip platform (63, 65), we tested whether they showed a similar distribution to that of the probes included on this array. We observed a significantly smaller proportion of DMRs around the gene promoters in cases with MUHO compared to controls. Whether or not this is a general feature of AT from MUHO subjects representing for example specific demethylation events at promoter regions or defects in targeting methylation to specific promoter regions, this will need to be established in multiple and larger cohorts. It will be important also to establish if the relative ‘loss’ of promoter DMRs has an impact on gene expression. Promoter DNA methylation is traditionally associated with repression of gene expression. Nonetheless, there are exceptions to this rule, and hypermethylation induced transcriptional activation has been document in a range of conditions and cell types (155). Although gene body methylation is highly conserved across eukaryotic species, the understanding of its function is still incomplete, and may play a role in alternative splicing or as a “fine tuning” mechanism of gene expression (156, 157). Because the data on gene expression in the studies included in this review was incomplete, we could not assess directly the relationship between the DMRs and gene expression. Nonetheless, we set out to perform bioinformatics analyses on the set of genes that associated DMRs above the cutoff of 5%. Both DAVID and IPA analyses highlighted alterations in several key intracellular signaling pathways. Among these, we identified a significant enrichment of genes implicated in signal transduction mediated by small GTPases. Several small GTPases, such as those from the Rho family, many of which associate DMRs in our study, have been already linked with glucose uptake in AT and with IR (158). Calcium homeostasis, enriched in genes associating DMRs in our study, has been also implicated in a myriad of cellular and subcellular dysfunctional networks found in the context of obesity and diabetes (159). ERK1/2 signaling (109, 111) and intracellular protein trafficking (101) were also linked previously with insulin signaling. Our search also identified a network of TFs predicted to regulate transcription of a large fraction of genes that associated DMRs and seven of these TFs were associated with DMRs themselves. While some of these TFs are strongly linked with glucose homeostasis, others, such as ELF1, IRF8 and RUNX1, are yet to be studied in this context. It has been reported that the binding site for ELF1 is overrepresented in the promoters of genes up-regulated during adipogenesis of human adipose-derived stromal cells (160). Both IRF8 (161–163) and RUNX1 (163) have been implicated in the pro-inflammatory immune response. It is known that AT inflammation plays an important role in the development of insulin resistance (3). Understanding the mechanisms by which the seven TFs identified in our analysis could target DNA methylation changes to specific regions requires further studies. Emerging evidence suggests that TF occupancy can mediate active turnover of DNA methylation by local recruitment of DNMT and TET enzymes implicated in the addition and erasure of DNA methyl groups, respectively (164, 165). As these TFs are involved in complex regulatory pathways and impact a great number of genes, more attention to their potential role in the pathogenesis of obesity and obesity-associated dysglycemia is warranted.

The search for genetic-based risk factors is particularly affected by the high degree of clinical heterogeneity that exists within each group of healthy or unhealthy individuals, but also because metabolic markers of ‘health’ that define these two groups vary greatly across current human studies and cohorts. In spite of these pitfalls, major insights into the identification of genetic pathways that may help uncoupling adiposity to cardiometabolic comorbidities have come from GWAS studies (32, 33). Huang et al. (33) identified 62 loci of which the adiposity-increasing allele was also associated with a favourable effect on cardiometabolic risk factors. Interestingly, we found that 10 of these genes (*ADCY9*, *ARAP1*, *CLIP1*, *CREBBP*, *GFI1*, *IRS1*, *JAZF1*, *NCOR2*, *PPARG* and *PSORS1C1*) are differentially methylated between subjects with MUHO and subjects with MHO in our systematic review lists. Another approach that may lead to the identification of gene variants that link increased adiposity with reduced risk of cardiometabolic is a more indirect one (32). Accordingly, GWAS studies aimed to discover genes involved in body fat % identified variants in or near *IRS1, COBLL1/GRB14, PLA2G6, TOMM40 loci* of which the body fat % increasing allele had protective effects on cardiometabolic outcomes. Interestingly, GWAS studies for obesity or T2D show relatively little overlap, with a screen using the GWAS Catalog (https://www.ebi.ac.uk/gwas/) leading to the identification of 89 gene variants common to both. Of these 89 genes, 8 (*SUGP1*, *MACROD2*, *RPTOR*, *FAIM2*, *LEPR*, *HS6ST3*, *TMEM1* and *KCNQ1*) were found differentially methylated between subjects with MUHO compared to subjects with MHO in our review lists.

Histone modifications are important epigenetic marks, with a regulatory role in numerous cellular events. These modifications can either promote or repress DNA transcription, and act through two main mechanisms, by affecting chromatin structure or protein binding (140, 166). Moreover, histone lysine methylation may activate or repress transcription depending on its position and methylation state (mono, di or trimethylation) (167). The scarcity of histone modifications studies in AT is related to technical difficulties. Indeed, the high lipid content renders AT as an extremely challenging tissue to work with. Clear associations were found between H3K4me3 enrichment in key genes and their expression (*E2F1, LPL, SREBF2, SCD, PPARG* and *IL6*) (71), illustrating that histone methylation could have an important role in obesity and T2D, by affecting various pathways associated with metabolic disease. The preliminary work discussed in this review indicate a great potential, but also highlight the need for genome-wide characterization of histone modifications in human AT to better understand their role in MUHO.

Unique lncRNA expression profiles in AT of individuals with obesity were found to correlate with distinct glycemic states (73, 74). These lncRNAs intervene in the glucose regulation pathways and are suggested to be implicated in the pathology of insulin resistance and T2D by modulating pathways involved in adipogenesis, energy metabolism, inflammation or insulin sensitivity (168, 169). *GAS5,* comprised of 12 exons, belongs to the 5’-terminal oligopyrimidine class and codifies not only the respective lncRNA, but also miRNAs, small nucleolar RNAs and PIWI-interacting RNAs (170, 171). The lncRNA transcript was suggested to interact with the promoter of the *INSR* gene as a riboactivator, allowing transcription factors to bind more easily, leading to overexpression of this gene (74). *GAS5* lncRNA was also previously reported to interact directly with the chromatin in other contexts, such as being a riborepressor of the glucocorticoid receptor in starvation or growth arrest (172). ENSG00000235609.4 and CATG00000111229.1 are novel intragenic lncRNAs and their role in the adipose physiology is largely unknown. Different AT depots and diferent cell populations may have specific patterns of lncRNA expression (173). Hence, it is critical to perform more studies on the role of differentially expressed lncRNAs in the different AT depots and on different cell populations comprised within the AT, in order to understand their functions. It is equally necessary to obtain more data on how these expression patterns vary with time and/or after clinical interventions, such as exercise, diet, drugs and even bariatric surgery, which could prove to be useful therapeutical targets for controling IR and T2D.

There are a few limitations to this systematic review that should be acknowledged. First, the number of studies characterizing epigenetic marks in the context of obesity-associated dysglycemia is very limited, and there are even fewer studies that used AT for such analyses. This limitation is particularly striking for studies on histone modifications and lncRNAs. Second, there is a great heterogeneity in patient characteristics analyzed across the studies, with high variability of anthropometric features, surrogate measurements used to define dysglycemia and different ethnic backgrounds. Third, the methodological approaches used by these studies were diverse and the magnitude of DNA methylation changes is overall small. Fourth and most importantly, gene expression was not assessed in the majority of these studies, hampering the possibility to evaluate the putative biological consequences of the DMRs.

In summary, the question on why some individuals with obesity are metabolically ‘healthy’, while others are metabolically ‘unhealthy’ is a complex one, mainly because there are a number of risk factors that have to be present or absent to define an individual as healthy or unhealthy. Those risk factors include environmental factors (e.g. physical activity, diet and smoking), demographic factors (e.g. sex, ancestry, age) as well as genetic factors. Based on our current analysis we propose that genome-wide epigenetic studies in AT have considerable promise as an additional approach to identify alleles that can be associated with protective and/or detrimental risk of IR and dysglycemic states, thereby contributing to the uncoupling of adiposity from its cardiometabolic comorbidities. Additionally, we propose that careful comparison between DNA methylation signatures in WB and AT in individuals with MUHO may lead to the development of new biomarkers with predictive power for progression of MHO towards metabolic complications. The advantage of using epigenetic marks for these studies is that they are powerful readouts of environmental influences on gene expression, thus potentially acting as links between genetic risk factors and environmental ones. However, the work performed in this systematic review also highlights the need to conduct more robust studies in AT of larger cohorts of individuals with MHO and MUHO. In addition, and to our knowledge, genome-wide transcriptome analyses have not been performed in AT in these two groups of interest, and epigenome screens at the level of regulatory elements, such as enhancers, are also missing. In our view, these are important future directions.

## Concluding Remarks

In summary, despite several major limitations, our systematic review led to the identification of a catalogue of epigenetic modifications, particularly DMRs, which highlighted several signaling pathways that are dysfunctional in AT of individuals with MUHO compared to AT of individuals that have MHO. Our approach also led to the identification of a network of TFs, some of which are novel in the context of AT biology. Additionally, we identified a small panel of individual CpGs that associate DNA methylation changes in WB and AT in individuals with MUHO versus MHO. Further studies are required to validate these findings and to assess whether they could lead to a better prediction of individuals with obesity at higher risk of developing T2D. This review also underscores the knowledge gap concerning histone modifications and lncRNA in obesity related-dysglycemia. Therefore, additional studies are needed, particularly those focusing on epigenetic mechanisms other than DNA methylation, and most importantly addressing how these affect gene expressions and dysregulate metabolic pathways leading to T2D.

## Data Availability Statement

The original contributions presented in the study are included in the article/**Supplementary Material**. Further inquiries can be directed to the corresponding author.

## Author Contributions

SA, ALS, and TM conducted the literature search, screened the papers, and drafted the manuscript. IS performed the bioinformatic analyses and drafted the manuscript. MC reviewed and edited the manuscript. MPM verified the literature search, conceived the research idea, and reviewed and edited the manuscript. All authors contributed to the article and approved the submitted version.

## Funding

This work is supported by the Portuguese Foundation for Science and Technology: “EPIADIPO - Exploring the role of epigenomic and adipocyte-associated signals in metabolic dysfunction” (PTDC/MEC-MET/32151/2017). Unit for Multidisciplinary Research in Biomedicine (UMIB) is funded by the Foundation for Science and Technology (FCT) Portugal (UIDB/00215/2020 and UIDP/00215/2020). TM has a grant from FCT, Portugal (SFRH/BD/123437/2016).

## Conflict of Interest

The authors declare that the research was conducted in the absence of any commercial or financial relationships that could be construed as a potential conflict of interest.

## References

[1] Fruhbeck G Busetto L Dicker D Yumuk V Goossens GH Hebebrand J. The ABCD of Obesity: An EASO Position Statement on a Diagnostic Term With Clinical and Scientific Implications. Obes Facts (2019) 12(2):131–6. 10.1159/000497124 PMC6547280 30844811

[2] Bremer AA Jialal I. Adipose Tissue Dysfunction in Nascent Metabolic Syndrome. J Obes (2013) 2013:393192. 10.1155/2013/393192 23653857 PMC3638696

[3] Zatterale F Longo M Naderi J Raciti GA Desiderio A Miele C. Chronic Adipose Tissue Inflammation Linking Obesity to Insulin Resistance and Type 2 Diabetes. Front Physiol (2019) 10:1607. 10.3389/fphys.2019.01607 32063863 PMC7000657

[4] Choe SS Huh JY Hwang IJ Kim JI Kim JB. Adipose Tissue Remodeling: Its Role in Energy Metabolism and Metabolic Disorders. Front Endocrinol (Lausanne) (2016) 7:30. 10.3389/fendo.2016.00030 27148161 PMC4829583

[5] Bergman M Abdul-Ghani M DeFronzo RA Manco M Sesti G Fiorentino TV. Review of Methods for Detecting Glycemic Disorders. Diabetes Res Clin Pract (2020) 165:108233. 10.1016/j.diabres.2020.108233 32497744 PMC7977482

[6] Kershaw EE Flier JS. Adipose Tissue as an Endocrine Organ. J Clin Endocrinol Metab (2004) 89(6):2548–56. 10.1210/jc.2004-0395 15181022

[7] DeFronzo RA. Pathogenesis of Type 2 Diabetes Mellitus. Med Clin North Am (2004) 88(4):787–835, ix. 10.1016/j.mcna.2004.04.013 15308380

[8] American Diabetes A. 2. Classification and Diagnosis of Diabetes: Standards of Medical Care in Diabetes-2019. Diabetes Care (2019) 42(Suppl 1):S13–28. 10.2337/dc19-S002 30559228

[9] Colditz GA Willett WC Rotnitzky A Manson JE. Weight Gain as a Risk Factor for Clinical Diabetes Mellitus in Women. Ann Intern Med (1995) 122(7):481–6. 10.7326/0003-4819-122-7-199504010-00001 7872581

[10] Guh DP Zhang W Bansback N Amarsi Z Birmingham CL Anis AH. The Incidence of Co-Morbidities Related to Obesity and Overweight: A Systematic Review and Meta-Analysis. BMC Public Health (2009) 9:88. 10.1186/1471-2458-9-88 19320986 PMC2667420

[11] Daousi C Casson IF Gill GV MacFarlane IA Wilding JP Pinkney JH. Prevalence of Obesity in Type 2 Diabetes in Secondary Care: Association With Cardiovascular Risk Factors. Postgrad Med J (2006) 82(966):280–4. 10.1136/pmj.2005.039032 PMC2579635 16597817

[12] Adams TD Davidson LE Litwin SE Kim J Kolotkin RL Nanjee MN. Weight and Metabolic Outcomes 12 Years After Gastric Bypass. N Engl J Med (2017) 377(12):1143–55. 10.1056/NEJMoa1700459 PMC5737957 28930514

[13] Caleyachetty R Thomas GN Toulis KA Mohammed N Gokhale KM Balachandran K. Metabolically Healthy Obese and Incident Cardiovascular Disease Events Among 3.5 Million Men and Women. J Am Coll Cardiol (2017) 70(12):1429–37. 10.1016/j.jacc.2017.07.763 28911506

[14] Prasad RB Groop L. Genetics of Type 2 Diabetes-Pitfalls and Possibilities. Genes (Basel) (2015) 6(1):87–123. 10.3390/genes6010087 25774817 PMC4377835

[15] Grarup N Sandholt CH Hansen T Pedersen O. Genetic Susceptibility to Type 2 Diabetes and Obesity: From Genome-Wide Association Studies to Rare Variants and Beyond. Diabetologia (2014) 57(8):1528–41. 10.1007/s00125-014-3270-4 24859358

[16] Voight BF Scott LJ Steinthorsdottir V Morris AP Dina C Welch RP. Twelve Type 2 Diabetes Susceptibility Loci Identified Through Large-Scale Association Analysis. Nat Genet (2010) 42(7):579–89. 10.1038/ng.609 PMC3080658 20581827

[17] Vujkovic M Keaton JM Lynch JA Miller DR Zhou J Tcheandjieu C. Discovery of 318 New Risk Loci for Type 2 Diabetes and Related Vascular Outcomes Among 1.4 Million Participants in a Multi-Ancestry Meta-Analysis. Nat Genet (2020) 52(7):680–91. 10.1101/19012690 PMC7343592 32541925

[18] Mahajan A Taliun D Thurner M Robertson NR Torres JM Rayner NW. Fine-Mapping Type 2 Diabetes Loci to Single-Variant Resolution Using High-Density Imputation and Islet-Specific Epigenome Maps. Nat Genet (2018) 50(11):1505–13. 10.1038/s41588-018-0241-6 PMC6287706 30297969

[19] Barroso I Luan J Middelberg RP Harding AH Franks PW Jakes RW. Candidate Gene Association Study in Type 2 Diabetes Indicates a Role for Genes Involved in Beta-Cell Function as Well as Insulin Action. PloS Biol (2003) 1(1):E20. 10.1371/journal.pbio.0000020 14551916 PMC212698

[20] Morris AP Voight BF Teslovich TM Ferreira T Segrè AV Steinthorsdottir V. Large-Scale Association Analysis Provides Insights Into the Genetic Architecture and Pathophysiology of Type 2 Diabetes. Nat Genet (2012) 44(9):981–90. 10.1038/ng.2383 PMC3442244 22885922

[21] Steinthorsdottir V Thorleifsson G Sulem P Helgason H Grarup N Sigurdsson A. Identification of Low-Frequency and Rare Sequence Variants Associated With Elevated or Reduced Risk of Type 2 Diabetes. Nat Genet (2014) 46(3):294–8. 10.1038/ng.2882 24464100

[22] Kooner JS Saleheen D Sim X Sehmi J Zhang W Frossard P. Genome-Wide Association Study in Individuals of South Asian Ancestry Identifies Six New Type 2 Diabetes Susceptibility Loci. Nat Genet (2011) 43(10):984–9. 10.1038/ng.921 PMC3773920 21874001

[23] Tong Y Lin Y Zhang Y Yang J Zhang Y Liu H. Association Between TCF7L2 Gene Polymorphisms and Susceptibility to Type 2 Diabetes Mellitus: A Large Human Genome Epidemiology (HuGE) Review and Meta-Analysis. BMC Med Genet (2009) 10:15. 10.1186/1471-2350-10-15 19228405 PMC2653476

[24] Xi C Miyaki K Ikeda S Song Y Sinbo T Muramatsu M. Association of GLUT4 Gene Variants With HbA1c Level in Japanese Men. Endocr J (2012) 59(8):677–84. 10.1507/endocrj.EJ11-0409 22673408

[25] Bains V Kaur H Badaruddoza B. Association Analysis of Polymorphisms in LEP (rs7799039 and rs2167270) and LEPR (rs1137101) Gene Towards the Development of Type 2 Diabetes in North Indian Punjabi Population. Gene (2020) 754:144846. 10.1016/j.gene.2020.144846 32512158

[26] Siitonen N Pulkkinen L Lindstrom J Kolehmainen M Eriksson JG Venojarvi M. Association of ADIPOQ Gene Variants With Body Weight, Type 2 Diabetes and Serum Adiponectin Concentrations: The Finnish Diabetes Prevention Study. BMC Med Genet (2011) 12:5. 10.1186/1471-2350-12-5 21219602 PMC3032655

[27] Ek J Andersen G Urhammer SA Gaede PH Drivsholm T Borch-Johnsen K. Mutation Analysis of Peroxisome Proliferator-Activated Receptor-Gamma Coactivator-1 (PGC-1) and Relationships of Identified Amino Acid Polymorphisms to Type II Diabetes Mellitus. Diabetologia (2001) 44(12):2220–6. 10.1007/s001250100032 11793024

[28] Grarup N Stender-Petersen KL Andersson EA Jorgensen T Borch-Johnsen K Sandbaek A. Association of Variants in the Sterol Regulatory Element-Binding Factor 1 (SREBF1) Gene With Type 2 Diabetes, Glycemia, and Insulin Resistance: A Study of 15,734 Danish Subjects. Diabetes (2008) 57(4):1136–42. 10.2337/db07-1534 18192539

[29] Hivert MF Manning AK McAteer JB Florez JC Dupuis J Fox CS. Common Variants in the Adiponectin Gene (ADIPOQ) Associated With Plasma Adiponectin Levels, Type 2 Diabetes, and Diabetes-Related Quantitative Traits: The Framingham Offspring Study. Diabetes (2008) 57(12):3353–9. 10.2337/db08-0700 PMC2584143 18776141

[30] Lall K Magi R Morris A Metspalu A Fischer K. Personalized Risk Prediction for Type 2 Diabetes: The Potential of Genetic Risk Scores. Genet Med (2017) 19(3):322–9. 10.1038/gim.2016.103 PMC5506454 27513194

[31] Lindstrom J Tuomilehto J. The Diabetes Risk Score: A Practical Tool to Predict Type 2 Diabetes Risk. Diabetes Care (2003) 26(3):725–31. 10.2337/diacare.26.3.725 12610029

[32] Loos RJF Kilpeläinen TO. Genes That Make You Fat, But Keep You Healthy. J Internal Med (2018) 284(5):450–63. 10.1111/joim.12827 PMC6566096 30144199

[33] Huang LO Rauch A Mazzaferro E Preuss M Carobbio S Bayrak CS. Genome-Wide Discovery of Genetic Loci That Uncouple Excess Adiposity From its Comorbidities. Nat Metab (2021) 3(2):228–43. 10.1038/s42255-021-00346-2 33619380

[34] Ling C Rönn T. Epigenetics in Human Obesity and Type 2 Diabetes. Cell Metab (2019) 29(5):1028–44. 10.1016/j.cmet.2019.03.009 PMC6509280 30982733

[35] Feinberg AP. The Key Role of Epigenetics in Human Disease Prevention and Mitigation. N Engl J Med (2018) 378(14):1323–34. 10.1056/NEJMra1402513 PMC11567374 29617578

[36] Feil R Fraga MF. Epigenetics and the Environment: Emerging Patterns and Implications. Nat Rev Genet (2012) 13(2):97–109. 10.1038/nrg3142 22215131

[37] Martinez JA Milagro FI Claycombe KJ Schalinske KL. Epigenetics in Adipose Tissue, Obesity, Weight Loss, and Diabetes. Adv Nutr (2014) 5(1):71–81. 10.3945/an.113.004705 24425725 PMC3884103

[38] Multhaup ML Seldin MM Jaffe AE Lei X Kirchner H Mondal P. Mouse-Human Experimental Epigenetic Analysis Unmasks Dietary Targets and Genetic Liability for Diabetic Phenotypes. Cell Metab (2015) 21(1):138–49. 10.1016/j.cmet.2014.12.014 PMC4340475 25565211

[39] Wahl S Drong A Lehne B Loh M Scott WR Kunze S. Epigenome-Wide Association Study of Body Mass Index, and the Adverse Outcomes of Adiposity. Nature (2017) 541(7635):81–6. 10.1038/nature20784 PMC5570525 28002404

[40] Chambers JC Loh M Lehne B Drong A Kriebel J Motta V. Epigenome-Wide Association of DNA Methylation Markers in Peripheral Blood From Indian Asians and Europeans With Incident Type 2 Diabetes: A Nested Case-Control Study. Lancet Diabetes Endocrinol (2015) 3(7):526–34. 10.1016/S2213-8587(15)00127-8 PMC4724884 26095709

[41] Ling C Del Guerra S Lupi R Rönn T Granhall C Luthman H. Epigenetic Regulation of PPARGC1A in Human Type 2 Diabetic Islets and Effect on Insulin Secretion. Diabetologia (2008) 51(4):615–22. 10.1007/s00125-007-0916-5 PMC2270364 18270681

[42] Barres R Kirchner H Rasmussen M Yan J Kantor FR Krook A. Weight Loss After Gastric Bypass Surgery in Human Obesity Remodels Promoter Methylation. Cell Rep (2013) 3(4):1020–7. 10.1016/j.celrep.2013.03.018 23583180

[43] Gillberg L Jacobsen SC Rönn T Brøns C Vaag A. Ppargc1a DNA Methylation in Subcutaneous Adipose Tissue in Low Birth Weight Subjects — Impact of 5days of High-Fat Overfeeding. Metabolism (2014) 63(2):263–71. 10.1016/j.metabol.2013.10.003 24262291

[44] Barrès R Osler ME Yan J Rune A Fritz T Caidahl K. Non-Cpg Methylation of the PGC-1α Promoter Through DNMT3B Controls Mitochondrial Density. Cell Metab (2009) 10(3):189–98. 10.1016/j.cmet.2009.07.011 19723495

[45] Barrès R Yan J Egan B Treebak JT Rasmussen M Fritz T. Acute Exercise Remodels Promoter Methylation in Human Skeletal Muscle. Cell Metab (2012) 15(3):405–11. 10.1016/j.cmet.2012.01.001 22405075

[46] Dayeh T Tuomi T Almgren P Perfilyev A Jansson PA de Mello VD. DNA Methylation of Loci Within ABCG1 and PHOSPHO1 in Blood DNA Is Associated With Future Type 2 Diabetes Risk. Epigenetics (2016) 11(7):482–8. 10.1080/15592294.2016.1178418 PMC4939923 27148772

[47] Loh M Zhou L Ng HK Chambers JC. Epigenetic Disturbances in Obesity and Diabetes: Epidemiological and Functional Insights. Mol Metab (2019) 27S:S33–41. 10.1016/j.molmet.2019.06.011 PMC6768506 31500829

[48] Walaszczyk E Luijten M Spijkerman AMW Bonder MJ Lutgers HL Snieder H. DNA Methylation Markers Associated With Type 2 Diabetes, Fasting Glucose and HbA(1c) Levels: A Systematic Review and Replication in a Case-Control Sample of the Lifelines Study. Diabetologia (2018) 61(2):354–68. 10.1007/s00125-017-4497-7 PMC6448925 29164275

[49] Einstein FH Atzmon G Yang XM Ma XH Rincon M Rudin E. Differential Responses of Visceral and Subcutaneous Fat Depots to Nutrients. Diabetes (2005) 54(3):672–8. 10.2337/diabetes.54.3.672 15734842

[50] Ibrahim MM. Subcutaneous and Visceral Adipose Tissue: Structural and Functional Differences. Obes Rev (2010) 11(1):11–8. 10.1111/j.1467-789X.2009.00623.x 19656312

[51] Wells GA Shea B O’Connell DA Peterson J Welch V Losos M. The Newcastle-Ottawa Scale (NOS) for Assessing the Quality of Nonrandomised Studies in Meta-Analyses. Oxford (2000).

[52] Castellano-Castillo D Moreno-Indias I Fernandez-Garcia JC Clemente-Postigo M Castro-Cabezas M Tinahones FJ. Complement Factor C3 Methylation and mRNA Expression Is Associated to BMI and Insulin Resistance in Obesity. Genes (Basel) (2018) 9(8):1–9. 10.3390/genes9080410 PMC6116013 30104553

[53] Houde AA Legare C Biron S Lescelleur O Biertho L Marceau S. Leptin and Adiponectin DNA Methylation Levels in Adipose Tissues and Blood Cells Are Associated With BMI, Waist Girth and LDL-cholesterol Levels in Severely Obese Men and Women. BMC Med Genet (2015) 16:29. 10.1186/s12881-015-0174-1 25929254 PMC4631085

[54] Krause C Sievert H Geissler C Grohs M El Gammal AT Wolter S. Critical Evaluation of the DNA-Methylation Markers ABCG1 and SREBF1 for Type 2 Diabetes Stratification. Epigenomics (2019) 11(8):885–97. 10.2217/epi-2018-0159 31169416

[55] Main AM Gillberg L Jacobsen AL Nilsson E Gjesing AP Hansen T. DNA Methylation and Gene Expression of HIF3A: Cross-Tissue Validation and Associations With BMI and Insulin Resistance. Clin Epigenet (2016) 8:89. 10.1186/s13148-016-0258-6 PMC5010678 27594926

[56] Malodobra-Mazur M Alama A Bednarska-Chabowska D Pawelka D Myszczyszyn A Dobosz T. Obesity-Induced Insulin Resistance *Via* Changes in the DNA Methylation Profile of Insulin Pathway Genes. Adv Clin Exp Med (2019) 28(12):1599–607. 10.17219/acem/110321 31766080

[57] Willmer T Goedecke JH Dias S Louw J Pheiffer C. DNA Methylation of FKBP5 in South African Women: Associations With Obesity and Insulin Resistance. Clin Epigenet (2020) 12(1):141. 10.1186/s13148-020-00932-3 PMC7507280 32958048

[58] You D Nilsson E Tenen DE Lyubetskaya A Lo JC Jiang R. Dnmt3a is an Epigenetic Mediator of Adipose Insulin Resistance. Elife (2017) 6:1–20. 10.7554/eLife.30766 PMC5730374 29091029

[59] Zhang J Wang C Ha X Li W Xu P Gu Y. DNA Methylation of Tumor Necrosis Factor-Alpha, Monocyte Chemoattractant Protein-1, and Adiponectin Genes in Visceral Adipose Tissue Is Related to Type 2 Diabetes in the Xinjiang Uygur Population. J Diabetes (2017) 9(7):699–706. 10.1111/1753-0407.12478 27573980

[60] Andersen E Ingerslev LR Fabre O Donkin I Altintas A Versteyhe S. Preadipocytes From Obese Humans With Type 2 Diabetes Are Epigenetically Reprogrammed at Genes Controlling Adipose Tissue Function. Int J Obes (Lond) (2019) 43(2):306–18. 10.1038/s41366-018-0031-3 29511320

[61] Arner P Sahlqvist AS Sinha I Xu H Yao X Waterworth D. The Epigenetic Signature of Systemic Insulin Resistance in Obese Women. Diabetologia (2016) 59(11):2393–405. 10.1007/s00125-016-4074-5 PMC5506095 27535281

[62] Barajas-Olmos F Centeno-Cruz F Zerrweck C Imaz-Rosshandler I Martinez-Hernandez A Cordova EJ. Altered DNA Methylation in Liver and Adipose Tissues Derived From Individuals With Obesity and Type 2 Diabetes. BMC Med Genet (2018) 19(1):28. 10.1186/s12881-018-0542-8 29466957 PMC5822594

[63] Crujeiras AB Diaz-Lagares A Moreno-Navarrete JM Sandoval J Hervas D Gomez A. Genome-Wide DNA Methylation Pattern in Visceral Adipose Tissue Differentiates Insulin-Resistant From Insulin-Sensitive Obese Subjects. Transl Res (2016) 178:13–24.e5. 10.1016/j.trsl.2016.07.002 27477082

[64] Lee K Moon S Park MJ Koh IU Choi NH Yu HY. Integrated Analysis of Tissue-Specific Promoter Methylation and Gene Expression Profile in Complex Diseases. Int J Mol Sci (2020) 21(14):1–16. 10.3390/ijms21145056 PMC7404266 32709145

[65] Nilsson E Jansson PA Perfilyev A Volkov P Pedersen M Svensson MK. Altered DNA Methylation and Differential Expression of Genes Influencing Metabolism and Inflammation in Adipose Tissue From Subjects With Type 2 Diabetes. Diabetes (2014) 63(9):2962–76. 10.2337/db13-1459 24812430

[66] Orozco LD Farrell C Hale C Rubbi L Rinaldi A Civelek M. Epigenome-Wide Association in Adipose Tissue From the METSIM Cohort. Hum Mol Genet (2018) 27(10):1830–46. 10.1093/hmg/ddy093 PMC5932563 29566149

[67] Pietilainen KH Ismail K Jarvinen E Heinonen S Tummers M Bollepalli S. DNA Methylation and Gene Expression Patterns in Adipose Tissue Differ Significantly Within Young Adult Monozygotic BMI-Discordant Twin Pairs. Int J Obes (Lond) (2016) 40(4):654–61. 10.1038/ijo.2015.221 26499446

[68] Ribel-Madsen R Fraga MF Jacobsen S Bork-Jensen J Lara E Calvanese V. Genome-Wide Analysis of DNA Methylation Differences in Muscle and Fat From Monozygotic Twins Discordant for Type 2 Diabetes. PloS One (2012) 7(12):e51302. 10.1371/journal.pone.0051302 23251491 PMC3519577

[69] Ronn T Volkov P Gillberg L Kokosar M Perfilyev A Jacobsen AL. Impact of Age, BMI and HbA1c Levels on the Genome-Wide DNA Methylation and mRNA Expression Patterns in Human Adipose Tissue and Identification of Epigenetic Biomarkers in Blood. Hum Mol Genet (2015) 24(13):3792–813. 10.1093/hmg/ddv124 25861810

[70] Wang C Ha X Li W Xu P Zhang Z Wang T. Comparative Gene Expression Profile and DNA Methylation Status in Diabetic Patients of Kazak and Han People. Med (Baltimore) (2018) 97(36):e11982. 10.1097/MD.0000000000011982 PMC6133596 30200077

[71] Castellano-Castillo D Denechaud PD Fajas L Moreno-Indias I Oliva-Olivera W Tinahones F. Human Adipose Tissue H3K4me3 Histone Mark in Adipogenic, Lipid Metabolism and Inflammatory Genes Is Positively Associated With BMI and HOMA-IR. PloS One (2019) 14(4):e0215083. 10.1371/journal.pone.0215083 30958852 PMC6453466

[72] Jufvas A Sjodin S Lundqvist K Amin R Vener AV Stralfors P. Global Differences in Specific Histone H3 Methylation Are Associated With Overweight and Type 2 Diabetes. Clin Epigenet (2013) 5(1):15. 10.1186/1868-7083-5-15 PMC3766271 24004477

[73] Gao H Kerr A Jiao H Hon CC Ryden M Dahlman I. Long Non-Coding RNAs Associated With Metabolic Traits in Human White Adipose Tissue. EBioMedicine (2018) 30:248–60. 10.1016/j.ebiom.2018.03.010 PMC5952343 29580841

[74] Shi Y Parag S Patel R Lui A Murr M Cai J. Stabilization of Lncrna GAS5 by a Small Molecule and Its Implications in Diabetic Adipocytes. Cell Chem Biol (2019) 26(3):319–30.e6. 10.1016/j.chembiol.2018.11.012 30661991 PMC10498384

[75] Supek F Bosnjak M Skunca N Smuc T. REVIGO Summarizes and Visualizes Long Lists of Gene Ontology Terms. PloS One (2011) 6(7):e21800. 10.1371/journal.pone.0021800 21789182 PMC3138752

[76] Andersen SL Briggs FBS Winnike JH Natanzon Y Maichle S Knagge KJ. Metabolome-Based Signature of Disease Pathology in MS. Mult Scler Relat Disord (2019) 31:12–21. 10.1016/j.msard.2019.03.006 30877925 PMC6548586

[77] Do C Lang CF Lin J Darbary H Krupska I Gaba A. Mechanisms and Disease Associations of Haplotype-Dependent Allele-Specific DNA Methylation. Am J Hum Genet (2016) 98(5):934–55. 10.1016/j.ajhg.2016.03.027 PMC4863666 27153397

[78] Bruehl S Gamazon ER Van de Ven T Buchheit T Walsh CG Mishra P. DNA Methylation Profiles Are Associated With Complex Regional Pain Syndrome After Traumatic Injury. Pain (2019) 160(10):2328–37. 10.1097/j.pain.0000000000001624 PMC7473388 31145213

[79] Storry JR Peyrard T. The Vel Blood Group System: A Review. Immunohematology (2017) 33(2):56–9. 10.21307/immunohematology-2019-008 28657763

[80] Jaafar L Chamseddine Z El-Sibai M. StarD13: A Potential Star Target for Tumor Therapeutics. Hum Cell (2020) 33(3):437–43. 10.1007/s13577-020-00358-2 32274657

[81] Alpy F Tomasetto C. START Ships Lipids Across Interorganelle Space. Biochimie (2014) 96:85–95. 10.1016/j.biochi.2013.09.015 24076129

[82] Naumann H Rathjen T Poy MN Spagnoli FM. The RhoGAP Stard13 Controls Insulin Secretion Through F-Actin Remodeling. Mol Metab (2018) 8:96–105. 10.1016/j.molmet.2017.12.013 29310936 PMC5985048

[83] Hatch GM Gu Y Xu FY Cizeau J Neumann S Park JS. Stard13(Dlc-2) RhoGap Mediates Ceramide Activation of Phosphatidylglycerolphosphate Synthase and Drug Response in Chinese Hamster Ovary Cells. Mol Biol Cell (2008) 19(3):1083–92. 10.1091/mbc.e06-08-0737 PMC2262983 18162584

[84] Li Y Talbot CL Chaurasia B. Ceramides in Adipose Tissue. Front Endocrinol (Lausanne) (2020) 11:407. 10.3389/fendo.2020.00407 32636806 PMC7316884

[85] Li X Liu B Ji CN Kang Y Mao Y. Cloning and Expression of ARMC3_v2, A Novel Splicing Variant of the Human ARMC3 Gene. Genetika (2006) 42(7):999–1003. 10.1134/S1022795406070209 16915934

[86] Abiola M Favier M Christodoulou-Vafeiadou E Pichard AL Martelly I Guillet-Deniau I. Activation of Wnt/beta-catenin Signaling Increases Insulin Sensitivity Through a Reciprocal Regulation of Wnt10b and SREBP-1c in Skeletal Muscle Cells. PloS One (2009) 4(12):e8509. 10.1371/journal.pone.0008509 20041157 PMC2794543

[87] Bilkovski R Schulte DM Oberhauser F Mauer J Hampel B Gutschow C. Adipose Tissue Macrophages Inhibit Adipogenesis of Mesenchymal Precursor Cells Via wnt-5a in Humans. Int J Obes (Lond) (2011) 35(11):1450–4. 10.1038/ijo.2011.6 21285942

[88] Figueroa KP Waters MF Garibyan V Bird TD Gomez CM Ranum LP. Frequency of KCNC3 DNA Variants as Causes of Spinocerebellar Ataxia 13 (SCA13). PloS One (2011) 6(3):e17811. 10.1371/journal.pone.0017811 21479265 PMC3066194

[89] You MH Song MS Lee SK Ryu PD Lee SY Kim DY. Voltage-Gated K+ Channels in Adipogenic Differentiation of Bone Marrow-Derived Human Mesenchymal Stem Cells. Acta Pharmacol Sin (2013) 34(1):129–36. 10.1038/aps.2012.142 PMC4086504 23222271

[90] Zhang Y Varela L Szigeti-Buck K Williams A Stoiljkovic M Sestan-Pesa M. Cerebellar Kv3.3 Potassium Channels Activate TANK-Binding Kinase 1 to Regulate Trafficking of the Cell Survival Protein Hax-1. Nat Commun (2021) 12(1):1731. 10.1038/s41467-021-22003-8 33741962 PMC7979925

[91] Zhao P Wong KI Sun X Reilly SM Uhm M Liao Z. TBK1 at the Crossroads of Inflammation and Energy Homeostasis in Adipose Tissue. Cell (2018) 172(4):731–43.e12. 10.1016/j.cell.2018.01.007 29425491 PMC5808582

[92] Kobayashi K Ogata H Morikawa M Iijima S Harada N Yoshida T. Distribution and Partial Characterisation of IgG Fc Binding Protein in Various Mucin Producing Cells and Body Fluids. Gut (2002) 51(2):169–76. 10.1136/gut.51.2.169 PMC1773314 12117874

[93] Gazi MH He M Cheville JC Young CY. Downregulation of IgG Fc Binding Protein (Fc gammaBP) in Prostate Cancer. Cancer Biol Ther (2008) 7(1):70–5. 10.4161/cbt.7.1.5131 17938577

[94] Ellulu MS Patimah I Khaza’ai H Rahmat A Abed Y. Obesity and Inflammation: The Linking Mechanism and the Complications. Arch Med Sci (2017) 13(4):851–63. 10.5114/aoms.2016.58928 PMC5507106 28721154

[95] Wang K Guan C Shang X Ying X Mei S Zhu H. A Bioinformatic Analysis: The Overexpression and Clinical Significance of FCGBP in Ovarian Cancer. Aging (Albany NY) (2021) 13(5):7416–29. 10.18632/aging.202601 PMC7993703 33686968

[96] Lavrsen K Dabelsteen S Vakhrushev SY Levann AMR Haue AD Dylander A. De Novo Expression of Human Polypeptide N-acetylgalactosaminyltransferase 6 (GalNAc-T6) in Colon Adenocarcinoma Inhibits the Differentiation of Colonic Epithelium. J Biol Chem (2018) 293(4):1298–314. 10.1074/jbc.M117.812826 PMC5787806 29187600

[97] Liesche F Kolbl AC Ilmer M Hutter S Jeschke U Andergassen U. Role of N-acetylgalactosaminyltransferase 6 in Early Tumorigenesis and Formation of Metastasis. Mol Med Rep (2016) 13(5):4309–14. 10.3892/mmr.2016.5044 27035742

[98] Chan PC Wu TN Chen YC Lu CH Wabitsch M Tian YF. Targetted Inhibition of CD74 Attenuates Adipose COX-2-MIF-Mediated M1 Macrophage Polarization and Retards Obesity-Related Adipose Tissue Inflammation and Insulin Resistance. Clin Sci (Lond) (2018) 132(14):1581–96. 10.1042/CS20180041 29773671

[99] Kang HS Liao G DeGraff LM Gerrish K Bortner CD Garantziotis S. CD44 Plays a Critical Role in Regulating Diet-Induced Adipose Inflammation, Hepatic Steatosis, and Insulin Resistance. PloS One (2013) 8(3):e58417. 10.1371/journal.pone.0058417 23505504 PMC3591334

[100] Niedick I Froese N Oumard A Mueller PP Nourbakhsh M Hauser H. Nucleolar Localization and Mobility Analysis of the NF-KappaB Repressing Factor NRF. J Cell Sci (2004) 117(Pt 16):3447–58. 10.1242/jcs.01129 15226370

[101] Samuel VT Shulman GI. Mechanisms for Insulin Resistance: Common Threads and Missing Links. Cell (2012) 148(5):852–71. 10.1016/j.cell.2012.02.017 PMC3294420 22385956

[102] Ahmed B Sultana R Greene MW. Adipose Tissue and Insulin Resistance in Obese. BioMed Pharmacother (2021) 137:111315. 10.1016/j.biopha.2021.111315 33561645

[103] Laurencikiene J van Harmelen V Arvidsson Nordstrom E Dicker A Blomqvist L Naslund E. NF-Kappab Is Important for TNF-Alpha-Induced Lipolysis in Human Adipocytes. J Lipid Res (2007) 48(5):1069–77. 10.1194/jlr.M600471-JLR200 17272828

[104] Berg AH Lin Y Lisanti MP Scherer PE. Adipocyte Differentiation Induces Dynamic Changes in NF-kappaB Expression and Activity. Am J Physiol Endocrinol Metab (2004) 287(6):E1178–88. 10.1152/ajpendo.00002.2004 15251865

[105] Vidhyasagar V He Y Guo M Talwar T Singh RS Yadav M. Biochemical Characterization of INTS3 and C9ORF80, Two Subunits of hNABP1/2 Heterotrimeric Complex in Nucleic Acid Binding. Biochem J (2018) 475(1):45–60. 10.1042/BCJ20170351 29150435 PMC5748837

[106] Spinozzi S Albini S Best H Richard I. Calpains for Dummies: What You Need to Know About the Calpain Family. Biochim Biophys Acta Proteins Proteom (2021) 1869(5):140616. 10.1016/j.bbapap.2021.140616 33545367

[107] Hata S Abe M Suzuki H Kitamura F Toyama-Sorimachi N Abe K. Calpain 8/nCL-2 and Calpain 9/nCL-4 Constitute an Active Protease Complex, G-calpain, Involved in Gastric Mucosal Defense. PloS Genet (2010) 6(7):e1001040. 10.1371/journal.pgen.1001040 20686710 PMC2912385

[108] Hata S Kitamura F Yamaguchi M Shitara H Murakami M Sorimachi H. A Gastrointestinal Calpain Complex, G-Calpain, Is a Heterodimer of CAPN8 and CAPN9 Calpain Isoforms, Which Play Catalytic and Regulatory Roles, Respectively. J Biol Chem (2016) 291(53):27313–22. 10.1074/jbc.M116.763912 PMC5207157 27881674

[109] Ozaki KI Awazu M Tamiya M Iwasaki Y Harada A Kugisaki S. Targeting the ERK Signaling Pathway as a Potential Treatment for Insulin Resistance and Type 2 Diabetes. Am J Physiol Endocrinol Metab (2016) 310(8):E643–E51. 10.1152/ajpendo.00445.2015 26860984

[110] Bost F Aouadi M Caron L Even P Belmonte N Prot M. The Extracellular Signal-Regulated Kinase Isoform ERK1 Is Specifically Required for *In Vitro* and *In Vivo* Adipogenesis. Diabetes (2005) 54(2):402–11. 10.2337/diabetes.54.2.402 15677498

[111] Jager J Corcelle V Gremeaux T Laurent K Waget A Pages G. Deficiency in the Extracellular Signal-Regulated Kinase 1 (ERK1) Protects Leptin-Deficient Mice From Insulin Resistance Without Affecting Obesity. Diabetologia (2011) 54(1):180–9. 10.1007/s00125-010-1944-0 20953578

[112] Sano H Peck GR Blachon S Lienhard GE. A Potential Link Between Insulin Signaling and GLUT4 Translocation: Association of Rab10-GTP With the Exocyst Subunit Exoc6/6b. Biochem Biophys Res Commun (2015) 465(3):601–5. 10.1016/j.bbrc.2015.08.069 PMC4564306 26299925

[113] von Blume J Alleaume AM Kienzle C Carreras-Sureda A Valverde M Malhotra V. Cab45 is Required for Ca(2+)-Dependent Secretory Cargo Sorting at the Trans-Golgi Network. J Cell Biol (2012) 199(7):1057–66. 10.1083/jcb.201207180 PMC3529532 23266954

[114] Chen F Sha M Wang Y Wu T Shan W Liu J. Transcription Factor Ets-1 Links Glucotoxicity to Pancreatic Beta Cell Dysfunction Through Inhibiting PDX-1 Expression in Rodent Models. Diabetologia (2016) 59(2):316–24. 10.1007/s00125-015-3805-3 26564177

[115] Li K Qiu C Sun P Liu DC Wu TJ Wang K. Ets1-Mediated Acetylation of FoxO1 Is Critical for Gluconeogenesis Regulation During Feed-Fast Cycles. Cell Rep (2019) 26(11):2998–3010.e5. 10.1016/j.celrep.2019.02.035 30865889

[116] Lin L Pang W Chen K Wang F Gengler J Sun Y. Adipocyte Expression of PU.1 Transcription Factor Causes Insulin Resistance Through Upregulation of Inflammatory Cytokine Gene Expression and ROS Production. Am J Physiol Endocrinol Metab (2012) 302(12):E1550–9. 10.1152/ajpendo.00462.2011 PMC3378156 22454293

[117] Lackey DE Reis FCG Isaac R Zapata RC El Ouarrat D Lee YS. Adipocyte PU.1 Knockout Promotes Insulin Sensitivity in HFD-Fed Obese Mice. Sci Rep (2019) 9(1):14779. 10.1038/s41598-019-51196-8 31611602 PMC6791934

[118] Tan CK Leuenberger N Tan MJ Yan YW Chen Y Kambadur R. Smad3 Deficiency in Mice Protects Against Insulin Resistance and Obesity Induced by a High-Fat Diet. Diabetes (2011) 60(2):464–76. 10.2337/db10-0801 PMC3028346 21270259

[119] Al Muftah WA Al-Shafai M Zaghlool SB Visconti A Tsai PC Kumar P. Epigenetic Associations of Type 2 Diabetes and BMI in an Arab Population. Clin Epigenet (2016) 8:13. 10.1186/s13148-016-0177-6 PMC4730771 26823690

[120] Albao DS Cutiongco-de la Paz EM Mercado ME Lirio A Mariano M Kim S. Methylation Changes in the Peripheral Blood of Filipinos With Type 2 Diabetes Suggest Spurious Transcription Initiation at TXNIP. Hum Mol Genet (2019) 28(24):4208–18. 10.1093/hmg/ddz262 31691802

[121] Arpón A Milagro FI Ramos-Lopez O Mansego ML Santos JL Riezu-Boj JI. Epigenome-Wide Association Study in Peripheral White Blood Cells Involving Insulin Resistance. Sci Rep (2019) 9(1):2445. 10.1038/s41598-019-38980-2 30792424 PMC6385280

[122] Cardona A Day FR Perry JRB Loh M Chu AY Lehne B. Epigenome-Wide Association Study of Incident Type 2 Diabetes in a British Population: EPIC-Norfolk Study. Diabetes (2019) 68(12):2315–26. 10.2337/db18-0290 PMC6868468 31506343

[123] Juvinao-Quintero DL Marioni RE Ochoa-Rosales C Russ TC Deary IJ van Meurs JBJ. DNA Methylation of Blood Cells Is Associated With Prevalent Type 2 Diabetes in a Meta-Analysis of Four European Cohorts. Clin Epigenet (2021) 13(1):40. 10.1186/s13148-021-01027-3 PMC7903628 33622391

[124] Kulkarni H Kos MZ Neary J Dyer TD Kent JW Jr. Goring HH. Novel Epigenetic Determinants of Type 2 Diabetes in Mexican-American Families. Hum Mol Genet (2015) 24(18):5330–44. 10.1093/hmg/ddv232 PMC4550817 26101197

[125] Meeks KAC Henneman P Venema A Addo J Bahendeka S Burr T. Epigenome-Wide Association Study in Whole Blood on Type 2 Diabetes Among Sub-Saharan African Individuals: Findings From the RODAM Study. Int J Epidemiol (2019) 48(1):58–70. 10.1093/ije/dyy171 30107520 PMC6380309

[126] Meeks KAC Henneman P Venema A Burr T Galbete C Danquah I. An Epigenome-Wide Association Study in Whole Blood of Measures of Adiposity Among Ghanaians: The RODAM Study. Clin Epigenet (2017) 9:103. 10.1186/s13148-017-0403-x PMC5609006 28947923

[127] Soriano-Tárraga C Jiménez-Conde J Giralt-Steinhauer E Mola-Caminal M Vivanco-Hidalgo RM Ois A. Epigenome-Wide Association Study Identifies TXNIP Gene Associated With Type 2 Diabetes Mellitus and Sustained Hyperglycemia. Hum Mol Genet (2016) 25(3):609–19. 10.1093/hmg/ddv493 26643952

[128] Toperoff G Aran D Kark JD Rosenberg M Dubnikov T Nissan B. Genome-Wide Survey Reveals Predisposing Diabetes Type 2-Related DNA Methylation Variations in Human Peripheral Blood. Hum Mol Genet (2012) 21(2):371–83. 10.1093/hmg/ddr472 PMC3276288 21994764

[129] Goodman KM Rubinstein R Thu CA Mannepalli S Bahna F Ahlsen G. Gamma-Protocadherin Structural Diversity and Functional Implications. Elife (2016) 5:1–25. 10.7554/eLife.20930 PMC5106212 27782885

[130] Kohmura N Senzaki K Hamada S Kai N Yasuda R Watanabe M. Diversity Revealed by a Novel Family of Cadherins Expressed in Neurons at a Synaptic Complex. Neuron (1998) 20(6):1137–51. 10.1016/S0896-6273(00)80495-X 9655502

[131] Demaurex N Grinstein S. Na+/H+ Antiport: Modulation by ATP and Role in Cell Volume Regulation. J Exp Biol (1994) 196:389–404. 10.1242/jeb.196.1.389 7823036

[132] Paehler Vor der Nolte A Chodisetti G Yuan Z Busch F Riederer B Luo M. Na(+) /H(+) Exchanger NHE1 and NHE2 Have Opposite Effects on Migration Velocity in Rat Gastric Surface Cells. J Cell Physiol (2017) 232(7):1669–80. 10.1002/jcp.25758 PMC5396337 28019659

[133] Stumpff F. A Look at the Smelly Side of Physiology: Transport of Short Chain Fatty Acids. Pflugers Arch (2018) 470(4):571–98. 10.1007/s00424-017-2105-9 29305650

[134] Salamone D Rivellese AA Vetrani C. The Relationship Between Gut Microbiota, Short-Chain Fatty Acids and Type 2 Diabetes Mellitus: The Possible Role of Dietary Fibre. Acta Diabetol (2021). 10.1007/s00592-021-01727-5 PMC8316221 33970303

[135] Chang HM Yeh ETH. Sumo: From Bench to Bedside. Physiol Rev (2020) 100(4):1599–619. 10.1152/physrev.00025.2019 PMC7717128 32666886

[136] Juliana CA Yang J Rozo AV Good A Groff DN Wang SZ. ATF5 Regulates Beta-Cell Survival During Stress. Proc Natl Acad Sci USA (2017) 114(6):1341–6. 10.1073/pnas.1620705114 PMC5307469 28115692

[137] Yuan Y Gaither K Kim E Liu E Hu M Lengel K. SUMO2/3 Modification of Activating Transcription Factor 5 (ATF5) Controls Its Dynamic Translocation at the Centrosome. J Biol Chem (2018) 293(8):2939–48. 10.1074/jbc.RA117.001151 PMC5827429 29326161

[138] Cassandri M Smirnov A Novelli F Pitolli C Agostini M Malewicz M. Zinc-Finger Proteins in Health and Disease. Cell Death Discovery (2017) 3:17071. 10.1038/cddiscovery.2017.71 29152378 PMC5683310

[139] Zhong C Chen C Yao F Fang W. ZNF251 Promotes the Progression of Lung Cancer by Activating ERK Signaling. Cancer Sci (2020) 111(9):3236–44. 10.1111/cas.14547 PMC7469813 32589309

[140] Hyun K Jeon J Park K Kim J. Writing, Erasing and Reading Histone Lysine Methylations. Exp Mol Med (2017) 49(4):e324. 10.1038/emm.2017.11 28450737 PMC6130214

[141] Statello L Guo CJ Chen LL Huarte M. Gene Regulation by Long non-Coding RNAs and Its Biological Functions. Nat Rev Mol Cell Biol (2021) 22(2):96–118. 10.1038/s41580-020-00315-9 33353982 PMC7754182

[142] Vigorelli V Resta J Bianchessi V Lauri A Bassetti B Agrifoglio M. Abnormal DNA Methylation Induced by Hyperglycemia Reduces CXCR4 Gene Expression in CD34+ Stem Cells. J Am Heart Assoc (2019) 8(9):e010012. 10.1161/JAHA.118.010012 31018749 PMC6512087

[143] Pepin ME Schiano C Miceli M Benincasa G Mansueto G Grimaldi V. The Human Aortic Endothelium Undergoes Dose-Dependent DNA Methylation in Response to Transient Hyperglycemia. Exp Cell Res (2021) 400(2):112485. 10.1016/j.yexcr.2021.112485 33515594 PMC8038422

[144] Menzaghi C Trischitta V Doria A. Genetic Influences of Adiponectin on Insulin Resistance, Type 2 Diabetes, and Cardiovascular Disease. Diabetes (2007) 56(5):1198–209. 10.2337/db06-0506 17303804

[145] Nieto-Vazquez I Fernández-Veledo S Krämer DK Vila-Bedmar R Garcia-Guerra L Lorenzo M. Insulin Resistance Associated to Obesity: The Link TNF-Alpha. Arch Physiol Biochem (2008) 114(3):183–94. 10.1080/13813450802181047 18629684

[146] Abel ED Peroni O Kim JK Kim YB Boss O Hadro E. Adipose-Selective Targeting of the GLUT4 Gene Impairs Insulin Action in Muscle and Liver. Nature (2001) 409(6821):729–33. 10.1038/35055575 11217863

[147] Feinberg AP Irizarry RA Fradin D Aryee MJ Murakami P Aspelund T. Personalized Epigenomic Signatures That Are Stable Over Time and Covary With Body Mass Index. Sci Transl Med (2010) 2(49):49ra67. 10.1126/scitranslmed.3001262 PMC3137242 20844285

[148] Elliott HR Shihab HA Lockett GA Holloway JW McRae AF Smith GD. Role of DNA Methylation in Type 2 Diabetes Etiology: Using Genotype as a Causal Anchor. Diabetes (2017) 66(6):1713–22. 10.2337/db16-0874 PMC5860189 28246294

[149] Rohde K Keller M la Cour Poulsen L Bluher M Kovacs P Bottcher Y. Genetics and Epigenetics in Obesity. Metabolism (2019) 92:37–50. 10.1016/j.metabol.2018.10.007 30399374

[150] Moltke I Grarup N Jorgensen ME Bjerregaard P Treebak JT Fumagalli M. A Common Greenlandic TBC1D4 Variant Confers Muscle Insulin Resistance and Type 2 Diabetes. Nature (2014) 512(7513):190–3. 10.1038/nature13425 25043022

[151] Thielen L Shalev A. Diabetes Pathogenic Mechanisms and Potential New Therapies Based Upon a Novel Target Called TXNIP. Curr Opin Endocrinol Diabetes Obes (2018) 25(2):75–80. 10.1097/MED.0000000000000391 29356688 PMC5831522

[152] Sharma PR Mackey AJ Dejene EA Ramadan JW Langefeld CD Palmer ND. An Islet-Targeted Genome-Wide Association Scan Identifies Novel Genes Implicated in Cytokine-Mediated Islet Stress in Type 2 Diabetes. Endocrinology (2015) 156(9):3147–56. 10.1210/en.2015-1203 PMC4541617 26018251

[153] Volkmar M Dedeurwaerder S Cunha DA Ndlovu MN Defrance M Deplus R. DNA Methylation Profiling Identifies Epigenetic Dysregulation in Pancreatic Islets From Type 2 Diabetic Patients. EMBO J (2012) 31(6):1405–26. 10.1038/emboj.2011.503 PMC3321176 22293752

[154] Noble JA Valdes AM. Genetics of the HLA Region in the Prediction of Type 1 Diabetes. Curr Diabetes Rep (2011) 11(6):533–42. 10.1007/s11892-011-0223-x PMC3233362 21912932

[155] Smith J Sen S Weeks RJ Eccles MR Chatterjee A. Promoter DNA Hypermethylation and Paradoxical Gene Activation. Trends Cancer (2020) 6(5):392–406. 10.1016/j.trecan.2020.02.007 32348735

[156] Lee SM Choi WY Lee J Kim YJ. The Regulatory Mechanisms of Intragenic DNA Methylation. Epigenomics (2015) 7(4):527–31. 10.2217/epi.15.38 26111026

[157] Greenberg MVC Bourc’his D. The Diverse Roles of DNA Methylation in Mammalian Development and Disease. Nat Rev Mol Cell Biol (2019) 20(10):590–607. 10.1038/s41580-019-0159-6 31399642

[158] Moller LLV Klip A Sylow L. Rho GTPases-Emerging Regulators of Glucose Homeostasis and Metabolic Health. Cells (2019) 8(5):1–21. 10.3390/cells8050434 PMC6562660 31075957

[159] Arruda AP Hotamisligil GS. Calcium Homeostasis and Organelle Function in the Pathogenesis of Obesity and Diabetes. Cell Metab (2015) 22(3):381–97. 10.1016/j.cmet.2015.06.010 PMC4558313 26190652

[160] Ambele MA Pepper MS. Identification of Transcription Factors Potentially Involved in Human Adipogenesis *In Vitro* . Mol Genet Genomic Med (2017) 5(3):210–22. 10.1002/mgg3.269 PMC5441431 28546992

[161] Sonne SB Yadav R Yin G Dalgaard MD Myrmel LS Gupta R. Obesity is Associated With Depot-Specific Alterations in Adipocyte DNA Methylation and Gene Expression. Adipocyte (2017) 6(2):124–33. 10.1080/21623945.2017.1320002 PMC5477735 28481699

[162] Pearl D Katsumura S Amiri M Tabatabaei N Zhang X Vinette V. 4e-Bp-Dependent Translational Control of Irf8 Mediates Adipose Tissue Macrophage Inflammatory Response. J Immunol (2020) 204(9):2392–400. 10.4049/jimmunol.1900538 32213561

[163] van Hamburg JP Tas SW. Molecular Mechanisms Underpinning T Helper 17 Cell Heterogeneity and Functions in Rheumatoid Arthritis. J Autoimmun (2018) 87:69–81. 10.1016/j.jaut.2017.12.006 29254845

[164] Suzuki T Maeda S Furuhata E Shimizu Y Nishimura H Kishima M. A Screening System to Identify Transcription Factors That Induce Binding Site-Directed DNA Demethylation. Epigenet Chromatin (2017) 10(1):60. 10.1186/s13072-017-0169-6 PMC5723091 29221486

[165] Ginno PA Gaidatzis D Feldmann A Hoerner L Imanci D Burger L. A Genome-Scale Map of DNA Methylation Turnover Identifies Site-Specific Dependencies of DNMT and TET Activity. Nat Commun (2020) 11(1):2680. 10.1038/s41467-020-16354-x 32471981 PMC7260214

[166] Bannister AJ Kouzarides T. Regulation of Chromatin by Histone Modifications. Cell Res (2011) 21(3):381–95. 10.1038/cr.2011.22 PMC3193420 21321607

[167] Cheung P Lau P. Epigenetic Regulation by Histone Methylation and Histone Variants. Mol Endocrinol (2005) 19(3):563–73. 10.1210/me.2004-0496 15677708

[168] Sathishkumar C Prabu P Mohan V Balasubramanyam M. Linking a Role of lncRNAs (Long Non-Coding RNAs) With Insulin Resistance, Accelerated Senescence, and Inflammation in Patients With Type 2 Diabetes. Hum Genomics (2018) 12(1):41. 10.1186/s40246-018-0173-3 30139387 PMC6107963

[169] Ruan Y Lin N Ma Q Chen R Zhang Z Wen W. Circulating LncRNAs Analysis in Patients With Type 2 Diabetes Reveals Novel Genes Influencing Glucose Metabolism and Islet Beta-Cell Function. Cell Physiol Biochem (2018) 46(1):335–50. 10.1159/000488434 29590649

[170] Coccia EM Cicala C Charlesworth A Ciccarelli C Rossi GB Philipson L. Regulation and Expression of a Growth Arrest-Specific Gene (gas5) During Growth, Differentiation, and Development. Mol Cell Biol (1992) 12(8):3514–21. 10.1128/MCB.12.8.3514 PMC364604 1630459

[171] Smith CM Steitz JA. Classification of Gas5 as a Multi-Small-Nucleolar-RNA (snoRNA) Host Gene and a Member of the 5’-Terminal Oligopyrimidine Gene Family Reveals Common Features of snoRNA Host Genes. Mol Cell Biol (1998) 18(12):6897–909. 10.1128/MCB.18.12.6897 PMC109273 9819378

[172] Kino T Hurt DE Ichijo T Nader N Chrousos GP. Noncoding RNA Gas5 Is a Growth Arrest- and Starvation-Associated Repressor of the Glucocorticoid Receptor. Sci Signal (2010) 3(107):ra8. 10.1126/scisignal.2000568 20124551 PMC2819218

[173] Jiang C Li Y Zhao Z Lu J Chen H Ding N. Identifying and Functionally Characterizing Tissue-Specific and Ubiquitously Expressed Human LncRNAs. Oncotarget (2016) 7(6):7120–33. 10.18632/oncotarget.6859 PMC4872773 26760768

