# Supplementary material for: Adipose Tissue Epigenetic Profile in Obesity-Related Dysglycemia - A Systematic Review
Source: Front Endocrinol (Lausanne). 2021 Jun 29;12:681649. doi: 10.3389/fendo.2021.681649 (PMC8288106; doi:10.3389/fendo.2021.681649)
Supplement: Supplementary file 1 [file DataSheet_1.docx]

**Supplementary Figure 1:** Common transcription factors between AME and IPA computational approaches that are predicted to regulate 149 of the genes in our dataset.

**Supplementary Table 1:** Search strategies for PubMed, Scopus, and Web of Science.

| PubMed |
| --- |
| (("type 2 diabetes" OR hyperglyc* OR dysglyc* OR "impaired glucose" OR "pre-diabetes" OR "Insulin Resistance") AND (epigen* OR *methylat* OR histones OR chromatin OR miRNA OR microRNA OR lncRNA OR long non-coding RNA)) AND adip* NOT ("cell line" OR cancer OR review OR animal OR gestational OR metformin OR "type 1 diabetes" OR "meta-analysis" OR "clinical trial" OR "stem cell" OR COVID-19)  ☒ Humans ☒ 10 years |
| Scopus |
| TITLE-ABS-KEY ( "type 2 diabetes" OR hyperglyc* OR dysglyc* OR "impaired glucose" OR pre-diabetes OR "Insulin Resistance" ) AND TITLE-ABS-KEY ( epigen* OR methylat OR histones OR chromatin* OR mirna OR microrna OR lncrna OR "long non-coding rna" ) AND TITLE-ABS-KEY ( adip* ) AND TITLE-ABS-KEY ( human ) AND NOT TITLE-ABS-KEY ( "cell line" OR cancer OR review OR animal OR gestational OR metformin OR "type 1 diabetes" OR meta-analysis OR "clinical trial" OR "stem cell" OR covid-19 ) AND ( LIMIT-TO ( PUBYEAR , 2020 ) OR LIMIT-TO ( PUBYEAR , 2019 ) OR LIMIT-TO ( PUBYEAR , 2018 ) OR LIMIT-TO ( PUBYEAR , 2017 ) OR LIMIT-TO ( PUBYEAR , 2016 ) OR LIMIT-TO ( PUBYEAR , 2015 ) OR LIMIT-TO ( PUBYEAR , 2014 ) OR LIMIT-TO ( PUBYEAR , 2013 ) OR LIMIT-TO ( PUBYEAR , 2012 ) OR LIMIT-TO ( PUBYEAR , 2011 ) OR LIMIT-TO ( PUBYEAR , 2010 ) ) |
| Web of Science |
| \|  \| #1:  ((TS=("type 2 diabetes" OR hyperglyc* OR dysglyc* OR "impaired glucose" OR pre-diabetes OR "Insulin Resistance") )  NOT  (TS=( "cell line" OR cancer OR review OR animal OR gestational OR metformin OR "type 1 diabetes" OR meta-analysis OR "clinical trial" OR "stem cell" OR covid-19) ))  *AND* **IDIOMA:**  (English)  *AND*  **TIPOS  DE  DOCUMENTO:**  (Article)  Índices=SCI-EXPANDED, SSCI, A&HCI, CPCI-S, CPCI-SSH, ESCI, CCR-EXPANDED, IC Tempo estipulado=2010-2020 \| \| --- \| --- \|   #2:   \|  \| (TS=(epigen* OR methylat* OR histones OR chromatin OR mirna OR microrna OR lncRNA OR “long non-coding RNA”)  AND  TS=(Human)  AND  TS=(Adip*))  *AND* **IDIOMA:**  (English)  *AND*  **TIPOS  DE  DOCUMENTO:**  (Article)  Índices=SCI-EXPANDED, SSCI, A&HCI, CPCI-S, CPCI-SSH, ESCI, CCR-EXPANDED, IC Tempo estipulado=2010-2020 \| \| --- \| --- \|   #3= #1 AND #2 |

Supplementary Table 2: Risk of Bias for cross-sectional studies with the Newcastle–Ottawa Quality Assessment Scale.

|  | Selection | Comparability | Outcome/Exposure | TOTAL |
| --- | --- | --- | --- | --- |
| Zhang, J. *et al*., 2017 | 4 | 1 | 3 | 8 |
| Houde, A.A. *et al*., 2015 | 3 | 2 | 3 | 8 |
| Main, A. M. *et al.*, 2016 | 4 | 1 | 3 | 8 |
| Willmer, T. *et al*., 2020 | 4 | 1 | 3 | 8 |
| Małodobra-Mazur, M. *et al.*, 2019 | 2 | 1 | 2 | 5 |
| You, D. *et al*., 2017 | 3 | 1 | 2 | 6 |
| Castellano-Castillo, D. *et al*., 2018 | 3 | 1 | 2 | 6 |
| Krause, C. *et al*., 2019 | 3 | 2 | 3 | 8 |
| Andersen, E. *et al.*, 2019 | 3 | 2 | 3 | 8 |
| Orozco L.D. *et al.*, 2018 | 2 | 1 | 3 | 6 |
| Lee, K. *et al*., 2020 | 2 | 2 | 3 | 7 |
| Wang, C. *et al*., 2018 | 2 | 2 | 2 | 6 |
| Crujeiras, A.B. *et al*., 2016 | 3 | 1 | 3 | 7 |
| Rönn, T. *et al.*, 2015 | 3 | 2 | 3 | 8 |
| Pietiläinen, K.H. *et al.*, 2016 | 4 | 1 | 3 | 8 |
| Nilsson, E *et al*., 2014 | 3 | 2 | 3 | 8 |
| Arner, P. *et al.*, 2016 | 4 | 2 | 3 | 9 |
| Barajas-Olmos, F *et al*., 2018 | 2 | 2 | 3 | 7 |
| Ribel-Madsen R. *et al*., 2012 | 3 | 2 | 3 | 8 |
| Jufvas A., *et al.*, 2013 | 1 | 2 | 2 | 5 |
| Castellano-Castillo D., *et al.*, 2019 | 3 | 2 | 3 | 8 |
| Gao, H. et al., 2018 | 4 | 1 | 3 | 8 |
| Shi, Y. *et al.,* 2019 | 1 | 2 | 2 | 5 |

**Supplementary Table 3:** Statistically significant DMRs present in the reviewed EWAS studies.

**Supplementary Table 4:** Genes that were differentially methylated by ≥5%, as well as their position within DAVID and REViGO clustering.

**Supplementary Table 5:** Genes that belong to enriched GO terms identified by DAVID analysis and are shown in Figure 3A, B, that have also been implicated in metabolic alterations.

| Gene | DNA methylation change (%) | Model | Metabolic alterations | Representative publication(s) |
| --- | --- | --- | --- | --- |
| *PAK3* | -18.6 | mouse | glucose intolerance under high fat diet | Piccand, J., *et al.*, 2014(1) |
| *PLSCR3* | -16.0 to -0.6 | mouse | insulin resistance, glucose intolerance and dyslipidemia | Wiedmer, T., *et al.*, 2004 (2) |
| *GNAS* | -10.0 to 3.4 | mouse, human | increased insulin sensitivity in knockout mice; gene variants associated with diabetes mellitus or insulin resistance in human | Yu, S., et al, 2001(3)  Yamamoto, M., *et al.,* 2004 (4)  Hahn, S., *et al.*, 2006 (5) |
| *S100A9* | -8.7 to -4.8 | mouse | insulin resistance | Xia, C., *et al.*, 2019 (6) |
| *FOXF2* | -7.0 to -3.0 | mouse | overexpression in adipose tissue associated with decrease glucose uptake *in vivo* | Westergren, R., *et al.*,2010 (7) |
| *RAPGEF1* | -7.2 to -3.6 | human | gene variants associated with T2D in Korean population | Hong, K.W., *et al.*,2009, (8) |
| *ARHGEF3* | -4.1 to 7.0 | human | genetic variant associated with increased insulin action in vivo | Kovacs, P., *et al.*,2006 (9) |
| *CHN2* | -4.0 to 21.2 | human | genetic variant associated with T2D in African Americans | Keaton, J.M., *et al.*, 2016 (10) |
| *STARD13* | 2.5 to 34.7 | mouse | controls insulin secretion | Naumann, H., *et al.*, 2018 (11) |
| *HLA-DRB1* | -17.0 to 55.0 | human | association with youth-onset type 1 diabetes in Pakistan and Bangladesh | Fawwad, A., *et al.*, 2019 (12)  Zabeen, B., *et al*.,2019 (13) |
| *HLA-DQA1* | 43.0 | human | glucose intolerance | Greenbaum, C.J., *et al.*, 2005 (14) |
| DNA methylation change reflects the range of change in individuals CpGs associated with the specific gene. A single value reflects a single CpG. | | | | |

**Supplementary Table 6:** Networks constructed via IPA analysis.

**Supplementary Table 7:** TFs motifs significantly enriched within sequences of DNA centered on DMRs found via AME, significantly enriched as upstream regulators of genes that associate DMRs uncovered via IPA analysis, and the overlap between the two.

Supplementary Table 8: CpGs with methylation changes above 5% in AT that were also found to be altered above 5% in WB

| TargetID | Gene Region | Gene | Whole Blood | | Adipose Tissue | | Concordant Direction |
| --- | --- | --- | --- | --- | --- | --- | --- |
|  |  |  | **Ref.** | **MUHO *vs* MHO (%)** | **Ref.** | **MUHO *vs* MHO (%)** |  |
| cg14642338 | TSS1500 | *PAMR1* | Barajas-Olmos, F. *et al.,* 2018 (15) | 5.0 | Barajas-Olmos, F. *et al.,* 2018 (15) | 7.0% | Hypermethylated |
| cg02707176 | Body | *PCDHGA1*/*PCDHGA4* | Arner, P. *et al.*, 2016 (16) | -5.9 | Crujeiras. A.B. *et al.*, 2016 (17) | -6.0 | Hypomethylated |
| cg09419670 | TSS1500; Body | *PSMD5* | Barajas-Olmos, F. *et al.,* 2018 (15) | 9.0 | Barajas-Olmos, F. *et al.,* 2018 (15) | 8.0 | Hypermethylated |
| cg20050113 | Body | *SLC9A2* | Barajas-Olmos, F. *et al.,* 2018 (15) | -5.0 | Barajas-Olmos, F. *et al.,* 2018 (15) | -9.0 | Hypomethylated |
| cg21053323 | Body | *SUMO3* | Barajas-Olmos, F. *et al.,* 2018 (15) | 6.0 | Barajas-Olmos, F. *et al.,* 2018 (15) | 6.0 | Hypermethylated |
| cg17878506 | TSS1500 | *TBC1D4* | Arner, P. *et al.*, 2016 (16) | 5.3 | Crujeiras. A.B. *et al.*, 2016 (17) | 12.0 | Hypermethylated |
| cg19693031 | 3′UTR | *TXNIP* | Soriano-Tárraga, C. *et al.,* 2016 (18) Kulkarni, H. *et al.*, 2015 (19) | -5.0 | Crujeiras. A.B. *et al.*, 2016 (17) | -7.0 | Hypomethylated |
| cg00117018 | Body | *ZNF251* | Arner, P. *et al.*, 2016 (16) | -6.1 | Crujeiras. A.B. *et al.*, 2016 (17) | -14.0 | Hypomethylated |
| cg07745373 | Body | *DTX4* | Arner, P. *et al.*, 2016 (16) | -6.0 | Crujeiras. A.B. *et al.*, 2016 (17) | 5.0 | No |
| cg13982505 | TSS1500 | *KCNJ13* | Barajas-Olmos, F. *et al.,* 2018 (15) | -6.0 | Barajas-Olmos, F. *et al.,* 2018 (15) | 5.0 | No |

1. Piccand J, Meunier A, Merle C, Jia Z, Barnier JV, Gradwohl G. Pak3 promotes cell cycle exit and differentiation of beta-cells in the embryonic pancreas and is necessary to maintain glucose homeostasis in adult mice. Diabetes. 2014;63(1):203-15.

2. Wiedmer T, Zhao J, Li L, Zhou Q, Hevener A, Olefsky JM, et al. Adiposity, dyslipidemia, and insulin resistance in mice with targeted deletion of phospholipid scramblase 3 (PLSCR3). Proc Natl Acad Sci U S A. 2004;101(36):13296-301.

3. Yu S, Castle A, Chen M, Lee R, Takeda K, Weinstein LS. Increased insulin sensitivity in Gsalpha knockout mice. J Biol Chem. 2001;276(23):19994-8.

4. Yamamoto M, Abe M, Jin JJ, Wu Z, Tabara Y, Mogi M, et al. Association of a GNAS1 gene variant with hypertension and diabetes mellitus. Hypertens Res. 2004;27(12):919-24.

5. Hahn S, Frey UH, Siffert W, Tan S, Mann K, Janssen OE. The CC genotype of the GNAS T393C polymorphism is associated with obesity and insulin resistance in women with polycystic ovary syndrome. Eur J Endocrinol. 2006;155(5):763-70.

6. Xia C, Razavi M, Rao X, Braunstein Z, Mao H, Toomey AC, et al. MRP14 enhances the ability of macrophage to recruit T cells and promotes obesity-induced insulin resistance. Int J Obes (Lond). 2019;43(12):2434-47.

7. Westergren R, Nilsson D, Heglind M, Arani Z, Grande M, Cederberg A, et al. Overexpression of Foxf2 in adipose tissue is associated with lower levels of IRS1 and decreased glucose uptake in vivo. Am J Physiol Endocrinol Metab. 2010;298(3):E548-54.

8. Hong KW, Jin HS, Lim JE, Ryu HJ, Go MJ, Lee JY, et al. RAPGEF1 gene variants associated with type 2 diabetes in the Korean population. Diabetes Res Clin Pract. 2009;84(2):117-22.

9. Kovacs P, Stumvoll M, Bogardus C, Hanson RL, Baier LJ. A functional Tyr1306Cys variant in LARG is associated with increased insulin action in vivo. Diabetes. 2006;55(5):1497-503.

10. Keaton JM, Hellwege JN, Ng MC, Palmer ND, Pankow JS, Fornage M, et al. Genome-Wide Interaction with Insulin Secretion Loci Reveals Novel Loci for Type 2 Diabetes in African Americans. PLoS One. 2016;11(7):e0159977.

11. Naumann H, Rathjen T, Poy MN, Spagnoli FM. The RhoGAP Stard13 controls insulin secretion through F-actin remodeling. Mol Metab. 2018;8:96-105.

12. Fawwad A, Govender D, Ahmedani MY, Basit A, Lane JA, Mack SJ, et al. Clinical features, biochemistry and HLA-DRB1 status in youth-onset type 1 diabetes in Pakistan. Diabetes Res Clin Pract. 2019;149:9-17.

13. Zabeen B, Govender D, Hassan Z, Noble JA, Lane JA, Mack SJ, et al. Clinical features, biochemistry and HLA-DRB1 status in children and adolescents with diabetes in Dhaka, Bangladesh. Diabetes Res Clin Pract. 2019;158:107894.

14. Greenbaum CJ, Eisenbarth G, Atkinson M, Yu L, Babu S, Schatz D, et al. High frequency of abnormal glucose tolerance in DQA1*0102/DQB1*0602 relatives identified as part of the Diabetes Prevention Trial--Type 1 Diabetes. Diabetologia. 2005;48(1):68-74.

15. Barajas-Olmos F, Centeno-Cruz F, Zerrweck C, Imaz-Rosshandler I, Martinez-Hernandez A, Cordova EJ, et al. Altered DNA methylation in liver and adipose tissues derived from individuals with obesity and type 2 diabetes. BMC Med Genet. 2018;19(1):28.

16. Arner P, Sahlqvist AS, Sinha I, Xu H, Yao X, Waterworth D, et al. The epigenetic signature of systemic insulin resistance in obese women. Diabetologia. 2016;59(11):2393-405.

17. Crujeiras AB, Diaz-Lagares A, Moreno-Navarrete JM, Sandoval J, Hervas D, Gomez A, et al. Genome-wide DNA methylation pattern in visceral adipose tissue differentiates insulin-resistant from insulin-sensitive obese subjects. Transl Res. 2016;178:13-24 e5.

18. Soriano-Tárraga C, Jiménez-Conde J, Giralt-Steinhauer E, Mola-Caminal M, Vivanco-Hidalgo RM, Ois A, et al. Epigenome-wide association study identifies TXNIP gene associated with type 2 diabetes mellitus and sustained hyperglycemia. Hum Mol Genet. 2016;25(3):609-19.

19. Kulkarni H, Kos MZ, Neary J, Dyer TD, Kent JW, Jr., Goring HH, et al. Novel epigenetic determinants of type 2 diabetes in Mexican-American families. Hum Mol Genet. 2015;24(18):5330-44.
