# Supplementary figures and images for: Adipose Tissue Epigenetic Profile in Obesity-Related Dysglycemia - A Systematic Review
Source: Front Endocrinol (Lausanne). 2021 Jun 29;12:681649. doi: 10.3389/fendo.2021.681649 (PMC8288106; doi:10.3389/fendo.2021.681649)

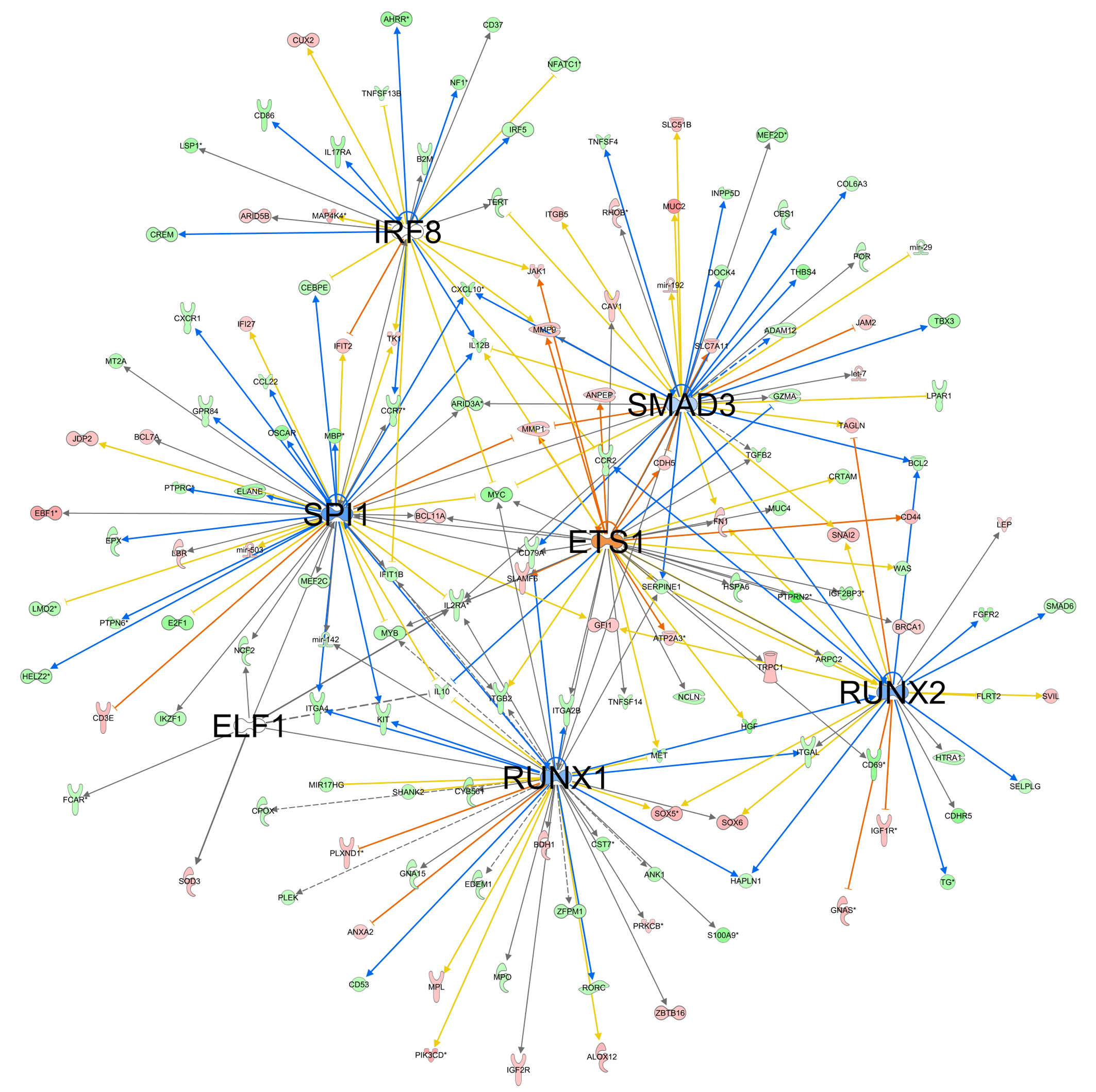

Supplement: Supplementary file 2 [file Image_1.tif]
